# UHRF1 establishes crosstalk between somatic and germ cells in male reproduction

**DOI:** 10.1038/s41419-022-04837-2

**Published:** 2022-04-19

**Authors:** Yanqing Wu, Peng Duan, Yujiao Wen, Jin Zhang, Xiaoli Wang, Juan Dong, Qiang Zhao, Shenglei Feng, Chunyu Lv, Yang Guo, Satoshi H. Namekawa, Shuiqiao Yuan

**Affiliations:** 1 Institute Reproductive Health, Tongji Medical College, Huazhong University of Science and Technology, Wuhan, Hubei 430030 China; 2 Laboratory of Gynecological Oncology and Reproductive Health, Department of Obstetrics and Gynaecology, Xiangyang No.1 People’s Hospital, Hubei University of Medicine, Xiangyang, Hubei 441000 China; 3 Hubei Clinical Research Center of Parkinson’s disease, Xiangyang Key Laboratory of Movement Disorders, Xiangyang No.1 People’s Hospital, Hubei University of Medicine, Xiangyang, Hubei 441000 China; 4 Department of Microbiology and Molecular Genetics, University of California Davis, Davis, CA 95616 USA; 5 Shenzhen Huazhong University of Science and Technology Research Institute, Shenzhen, Guangdong 518057 China

**Keywords:** Spermatogenesis, Infertility

## Abstract

Sertoli cells (SCs) support and nourish germ cells (GCs) through their crosstalk during spermatogenesis. However, the underlying epigenetic mechanism that ensures SCs’ functions in this process remains unclear. Here, we report that UHRF1, a critical epigenetic regulator, is mainly expressed in human and mouse pre-mature SCs, and is essential for establishing Sertoli-Germ cell crosstalk. SC-specific UHRF1 knockout mice exhibit complete sterility with Sertoli cell (SC) proliferation and differentiation aberrance, blood-testis barrier (BTB) disruption, and immature germ cell (GC) sloughing. RNA sequencing and Whole Genome Bisulfite Sequencing (WGBS) revealed that many extracellular matrix (ECM)-related genes (e.g., *Timp1*, *Trf*, and *Spp1*) appeared upregulated with the DNA hypomethylation status in UHRF1-deficient SCs. Strikingly, overexpression of *Timp1*, *Trf*, and *Spp1* in SCs in vitro and in vivo could phenocopy the SC-specific UHRF1-deficient mice. Our data demonstrated that UHRF1 regulates the transcriptional program of ECM-related genes in SCs and establishes SC-GC crosstalk.

## Introduction

In mammals, spermatogenesis is ensured via crosstalk between germ cells (GCs) and supporting somatic cells, Sertoli cells (SCs), in testes. SCs support and nourish GCs throughout the developmental stages in testes [1–4]. From the fetus to the juvenile, SCs provide nutrients, structural support, and waste-cleanup to facilitate male GC development and undergo gradual terminal differentiation at puberty to eventually support adult GCs [5–8]. During testicular development, SCs undergo proliferation, wherein the number of SCs increases until postnatal days 10 to 15 (P10-15), when SCs gradually cease proliferation and differentiate into mature adult SCs [9, 10]. The lifelong interactions between SCs and GCs are essential for germ cell (GC) development, differentiation, and maturation [11, 12].

During the proliferation and differentiation processes, SCs display some unique epigenetic features that may be associated with Sertoli cell (SC)-specific gene expression. First, SCs have a distinct heterochromatin structure that is highly methylated [13]. Second, in juvenile and adult rat SCs, 5-Hydroxymethylcytosine (5hmC), an intermediate of active DNA demethylation usually associated with repressive chromatin [14], is lost over the genes involved in cell adhesion, cytoskeleton, and cell morphology. However, the epigenetic mechanism that controls SC proliferation, differentiation, and SC-GC crosstalk remains unknown.

UHRF1, also known as ICBP90 in humans and NP95 in mice, is an important epigenetic regulator with multiple functional domains [15–17]. It contributes to the maintenance of DNA methylation and heterochromatin organization and regulates gene transcription and the ubiquitination of target proteins [18–20]. Early studies showed that UHRF1 is required for early development, and the global knockout of UHRF1 caused initial developmental arrest shortly after gastrulation in mice [21, 22]. We and others recently reported that UHRF1 is essential for germ cell development in both males and females by regulating several epigenetic pathways [23–28]. Despite the emerging functions of UHRF1 in GCs, it remains unknown whether UHRF1 is important for SC development.

Herein, we show that UHRF1 is specifically expressed in both human and mouse pre-mature SCs and essential for SC proliferation and differentiation during spermatogenesis. We discovered a critical role of UHRF1 in mediating SC cytoskeletal organization and SC-GC adhesion by maintaining CG methylation of ECM- and cell adhesion-related genes in SCs, as *Timp1, Trf, and Spp1*. Our data uncovered unexpected functions of UHRF1 for regulating SC-GC communications and SC dynamic cytoskeleton reorganization during spermatogenesis, with significant implications for male fertility.

## Results

### UHRF1 displays differential temporal and spatial expression patterns in SCs

**Fig. 1:**
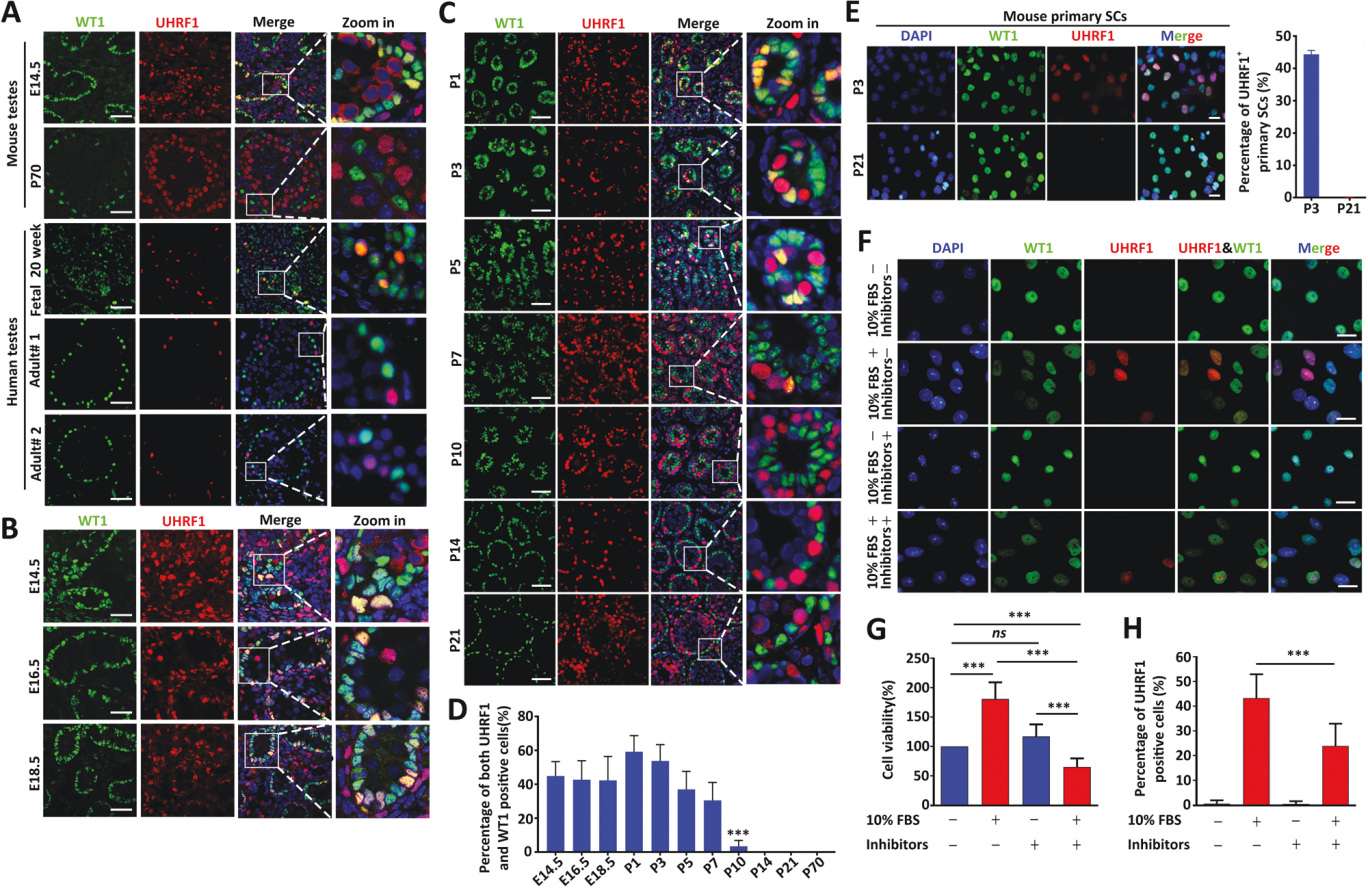
UHRF1 displays a temporal and spatial expression pattern in SCs of both humans and mice. **A** Double immunostaining with anti-UHRF1 and anti-WT1 antibodies on wild-type (WT) mouse testicular sections at embryonic day 14.5 (E14.5) and postnatal day 70 (P70) and human testis sections at fetal 20 weeks and adults are shown. Scale bar = 50μm. **B**, **C** Co-immunofluorescent staining for UHRF1 and WT1 (a SC marker) on WT mouse testis sections at E14.5, E16.5, E18.5, P1, P3, P5, P7, P10, P14, and P21 are shown. Nuclei were stained with DAPI. Scale bar = 50 μm. **D** The quantifications of the ratio of both UHRF1^+^ and WT1^+^ cells to WT1^+^ cells per tubule for **B** and **C** are shown. Data are shown the mean values ± SEM. For E14.5, E16.5, and E18.5, *n* = 3 mice; For P1, P3, P5, P7, and P10, *n* = 5 mice; For P14, P21, and P70, *n* = 7 mice. Approximately 20–30 round seminiferous tubules per mouse were randomly chosen to count. ***P < 0.001 by one-way ANOVA. **E** Double immunostaining with anti-UHRF1 and anti-WT1 on isolated primary SCs from P3 and P21 WT testes are shown. Scale bar = 20μm. Right histogram shows the percentage of both UHRF1^+^ and WT1^+^ cells to WT1^+^ cells. Data show mean values ± SEM, *n* = 3 mice. Approximately 30 round seminiferous tubules per mouse were randomly chosen to count. **F** Double immunostaining with UHRF1 and WT1 on primary SCs from P21 testes cultured with or without stem cell differentiation inhibitors (1 μM PD0325901 + 10 μM SP600125 + 10 μM SB203580) and 10% FBS for 24 h are shown. Scale bar = 20 μm. **G**, **H** Quantification of the percentage of UHRF1-positive cells (**G**) and cell viability (**H**) are shown. *ns* not significant. ***P < 0.001 by unpaired Student’s *t-*test.

To explore the functions of UHRF1 in SCs, we first analyzed its expression pattern in SCs at different developmental stages via immunostaining assays. We observed UHRF1 was highly expressed in pre-mature SCs with low levels in mature SCs, both in mouse and human testes (Fig. 1A). In mice, there was a gradual decline in UHRF1 levels in SCs from P1 to P14, at which point it was barely detected (Fig. 1B–D). Interestingly, we did not observe any decreases in the percentage of UHRF1-positive SCs from embryonic day (E18.5) to postnatal day (P1) (Fig. 1D). Further immunofluorescence analyses of purified mouse primary SCs showed that UHRF1 expression decreased from pre-mature (P3) to matured SCs (P21); specifically, we observed a decrease in the percentage of UHRF1-positive primary SCs from ~46% at P3 to ~1% at P21 (Fig. 1E), confirming that UHRF1 was expressed in pre-mature but not in mature SCs. Interestingly, the culture of primary SCs from P21 testes with medium supplemented with 10% (v/v) FBS, which facilitates proliferation, resulted in re-expression of UHRF1. The percentage of UHRF1-positive SCs was significantly higher in FBS-treated groups compared to controls (Fig. 1F, G and Supplementary Fig. 1A, B). Furthermore, pre-treatment of the SCs with stem cell differentiation inhibitors significantly attenuated FBS-induced elevation of cell viability and increased the UHRF1-positive cell ratio (Fig. 1G, H). These results suggest that UHRF1 is involved in SC proliferation and differentiation during development.

### UHRF1 in SCs is essential for spermatogenesis and male fertility

**Fig. 2:**
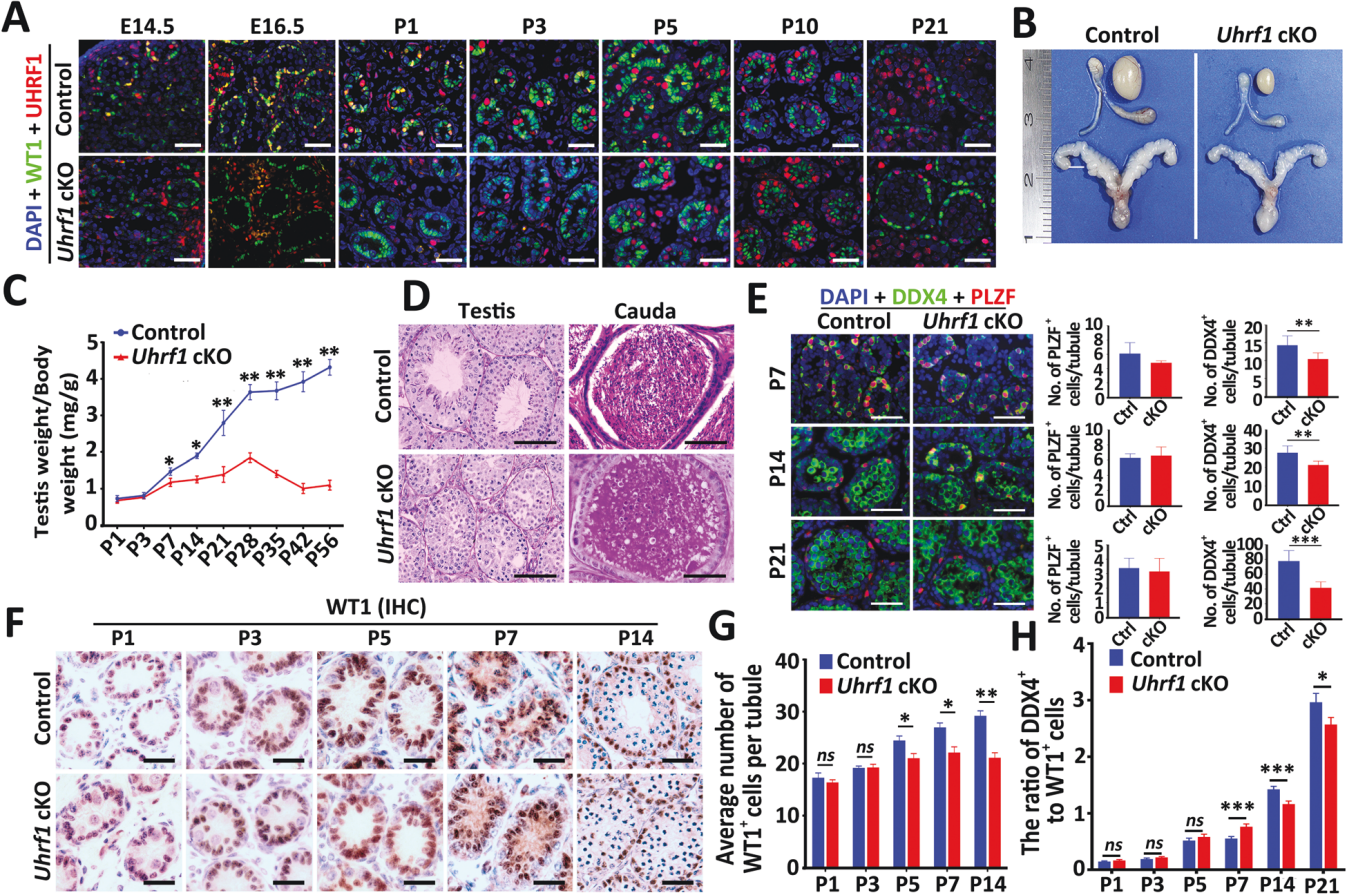
Conditional inactivation of *Uhrf1* in SCs results in spermatogenic arrest and male infertility in mice. **A** Representative co-immunofluorescent staining images for the UHRF1 and WT1 on control and *Uhrf1* cKO testes from E14.5, E16.5, P1, P3, P5, P10, and P21 mice are shown. Scale bar = 50 μm. Nuclei were stained with DAPI. **B** Gross morphology of the testis, epididymis, and seminal vesicle from control and *Uhrf1* cKO mice at P56. **C** Testis growth curve shows that the *Uhrf1* cKO testes were significantly decreased from P7. Data are presented as mean ± SEM, *n* = 3 mice. *P < 0.05, **P < 0.01 by unpaired Student’s *t-*test. **D** Periodic acid-Schiff (PAS) staining showing the histology of testis and cauda epididymis sections from control and *Uhrf1* cKO mice at P56. Scale bar = 100μm. **E** Co-immunofluorescent staining of DDX4 and PLZF in testis sections from control and *Uhrf1* cKO mice at P7, P14, and P21 is shown. Nuclei were stained with DAPI. Scale bar = 50 μm. The right histograms are showing the quantifications of PLZF^+^ cells per tubule and DDX4^+^ cells per tubule at P7, P14, and P21 in control and *Uhrf1* cKO mice. Data are presented as mean ± SEM. *n* = 7 mice for P3; n = 3 mice for P14 and P21. Approximately 30 round seminiferous tubules per mouse were randomly chosen to count. **P < 0.01, ***P < 0.001 by unpaired Student’s *t-*test. **F** Immunohistochemical staining of WT1 (a SC marker) in the developmental testis sections from control and *Uhrf1* cKO mice at P1, P3, P5, P7, and P14 is shown. Scale bar = 50 μm. **G** Histogram showing the quantification of WT1-positive cell numbers per tubule in **F**. Data are presented as mean ± SEM. For P1, P3, and P5, *n* = 5 mice. For P7 and P14, *n* = 3 mice. Approximately 10 round seminiferous tubules per mouse were randomly chosen to count. *ns*, not significant. *P < 0.05, **P < 0.01 by unpaired Student’s *t-*test. **H** Histogram showing the ratio of DDX4^+^ to WT1^+^ cell numbers per tubule in Supplementary Fig. 2e. For P1, P3, and P5, *n* = 5 mice. For P7, P14 and P21, *n* = 3 mice. Approximately 30 round seminiferous tubules per mouse were randomly chosen to count. Data are presented as mean ± SEM. *ns* not significant. *P < 0.05, **P < 0.01, ***P < 0.001 by unpaired Student’s *t-*test.

To understand the physiological role of UHRF1 in SCs during spermatogenesis, we generated SC-specific *Uhrf1* conditional knockout (cKO) mice to delete UHRF1 in pre-mature SCs using *Amh-*Cre line [29] (Supplementary Fig. 1C). Mice of genotype *Amh-Cre;Uhrf1*^*flox/flox*^ are herein designated *Uhrf1* cKO, and controls refer to age-matched *Uhrf1*^*flox/flox*^ or wild-type (WT) mice. Co-staining of UHRF1 with WT1 (a SC marker) in *Uhrf1* cKO and control testes from E14.5 to P21 confirmed complete UHRF1 depletion in SCs of testes (Fig. 2A). Adult *Uhrf1* cKO mice did not exhibit apparent physical abnormalities but were completely infertile with the testis and epididymis reduced in size (Fig. 2B, C). Histological analysis of testes and epididymides from adult *Uhrf1* cKO mice revealed notable degeneration and atrophied seminiferous tubules with the spermatogenic arrest at elongating/elongated spermatid, and many apoptotic GCs were observed in the cauda epididymis (Fig. 2D and Supplementary Fig. 2A–D). Moreover, we found that the number of PLZF^+^ or STRA8^+^ cells per seminiferous tubule were both comparable between *Uhrf1* cKO and control testes at P7, P14, and P21, while the number of DDX4^+^ GCs per seminiferous tubule decreased in *Uhrf1* cKO testes (Fig. 2E and Supplementary Fig. 3A). We also found that the number of WT1^+^ cells per seminiferous tubule significantly decreased in *Uhrf1* cKO testes compared with controls from P5 to P14 (Fig. 2F, G). Interestingly, the ratio of DDX4^+^ to WT1^+^ cell numbers per seminiferous tubule increased in *Uhrf1* cKO testis at P7 but decreased from P14 (Fig. 2H and Supplementary Fig. 2E). We then analyzed the ploidy of cells in the digested testis at P21 by flow cytometry. The results showed that the ratio of 1 N cell fractions (spermatids), 2 N fractions (spermatogonia and SCs), and 4 N cell fractions (mitotic spermatogonia and spermatocytes) had no significant change between control and *Uhrf1* cKO mice (Supplementary Fig. 2F, G). These data indicated that GCs are lost, but spermatogonial differentiation and meiotic processes appeared to be unaffected in *Uhrf1* cKO testes before P21. TUNEL assays further revealed that the number of TUNEL-positive cells was markedly increased in *Uhrf1* cKO testes compared to that of controls (Supplementary Fig. 3B, C). Together, these data demonstrated that, upon the deletion of UHRF1 in SCs, spermatogenic cells were gradually lost due to apoptosis from P7, eventually leading to spermatogenesis arrest at the elongating/elongated spermatid stage and male sterility.

### UHRF1 is required for SC proliferation and differentiation

To determine whether the damage of spermatogenesis in *Uhrf1* cKO mice is caused by abnormal proliferation and differentiation of SCs, we examined the proliferation and differentiation of SCs. We found that the number of SCs per seminiferous tubule significantly decreased in *Uhrf1* cKO testes compared with controls from P5 onwards (Fig. 2F, G and Supplementary Fig. 4A, B). Of note, unlike the consistent basal localization seen in control SCs, *Uhrf1* cKO SC nuclei (WT1^+^ cells) at P14 to P56 were found in the centers of >60% of the seminiferous tubules, ranging from lone nuclei to intratubular clusters of 5–10 nuclei (Supplementary Fig. 4A), suggesting the polarity of SCs were affected in *Uhrf1* cKO testes.

**Fig. 3:**
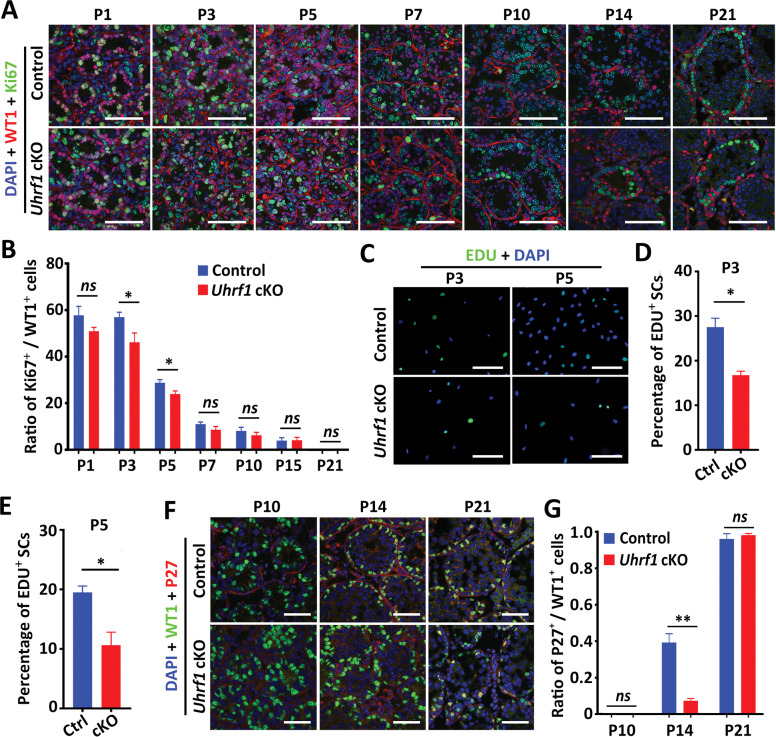
UHRF1 deficient SCs exhibit abnormal cell proliferation and differentiation. **A** Double immunofluorescent staining of WT1 (red) and Ki67 (green) is shown in both control and *Uhrf1* cKO mice at various ages. Nuclei were stained with DAPI. Scale bar =100μm. **B** The histogram shows that the quantification of the ratio of Ki67^+^ cells to WT1^+^ cells per tubule at P1, P3, P5, P7, P10, P14, and P21 in control and *Uhrf1* cKO testes. Data are presented as mean ± SD. For P1, P3, P5 and P7, *n* = 7 mice. For P14 and P21, *n* = 3 mice. Approximately 30 round seminiferous tubules per mouse were randomly chosen to count. *ns*, not significant. *P < 0.05 by unpaired Student’s *t*-test. **C** Fluorescent staining of EDU in primary SCs isolated from control and *Uhrf1* cKO testes at P3 and P5 is shown. Scale bar =50μm. **D, E** Histograms show the percentage of EDU^+^ SCs of (**D**) at P3 (**F**) and P5 (**E**). Data are presented as mean ± SEM. *n* = 5. *P < 0.05 by unpaired Student’s *t*-test. **F** Immunofluorescent staining for WT1 (green), P27 (Red), and DAPI (blue) on testis sections from control and *Uhrf1* cKO mice at P10, P14, and P21. Scale bar = 50μm. **G** Quantitation of the ratio of P27^+^ cells to WT1^+^ cells are shown from **F**. Data are presented as mean ± SEM, *n* = 3 mice. Approximately 30–50 round seminiferous tubules per mouse were randomly chosen to count. *ns* not significant. **P < 0.01 by unpaired Student’s *t-*test.

The proliferative activity of SCs was assessed by Ki-67 immunostaining. As shown in Fig. 3A, B, the *Uhrf1* cKO testes had a lower proportion of proliferative SCs at P3 and P5 in comparison to controls (*P* < 0.05). To further determine whether UHRF1 deficiency in SCs impedes the proliferation of SCs in vitro, we used EDU labeling to identify the S phase of SCs isolated from the control and *Uhrf1* cKO mice. The results showed that the percentage of EDU-positive SCs in *Uhrf1* cKO at both P3 and P5 was significantly less than that of the control (Fig. 3C–E). The protein P27 is a well-documented negative regulator of cell cycle progression [30]. Immunostaining of P27 assays revealed the ratio of P27^+^ SCs to total SCs in *Uhrf1* cKO testes at P14 was much lower than those of controls (Fig. 3F, G), suggesting a delay in initiating cell cycle arrest in *Uhrf1* cKO testes. Interestingly, the TUNEL assay showed that there is no significant difference in the number of apoptotic SCs between control and *Uhrf1* cKO testes at P21 and P43 (Supplementary Fig. 4C). Together, these results suggest that UHRF1 plays a pivotal role in the regulation of cell proliferation and cell cycle progression in SCs.

### Ablation of UHRF1 results in disruption of cytoskeletal structure in SCs

**Fig. 4:**
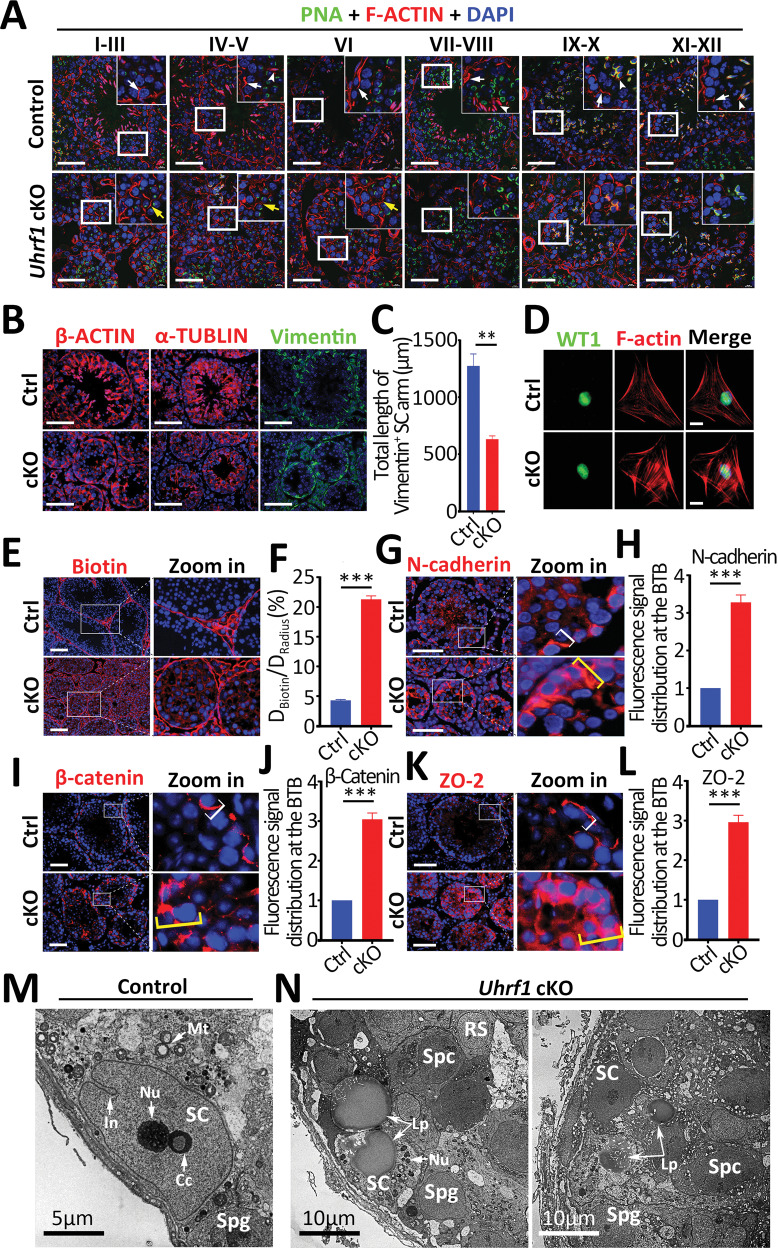
Ablation UHRF1 in SCs results in disorganization of SC actin cytoskeleton and severe disruption of the BTB. **A** Co-immunofluorescent staining with PNA (peanut agglutinin, green) and F-ACTIN (phalloidin, red) on different testicular stage sections from control and *Uhrf1* cKO mice at P42 is shown. Nuclei were stained with DAPI. Scale bar = 50μm. The right corner is an enlarged inset image. White arrows indicate basal ES, white arrowheads indicate apical ES, and yellow arrows indicate ectopically expressed F-ACTIN. **B** Immunofluorescence of β-actin, α-tubulin, and Vimentin shows that the SC polarity and cytoplasmic arms in the testes of control and *Uhrf1* cKO mice at P60. Scale bar = 50μm. **C** Quantification of Vimentin-positive cell arms in **B**. The total length of the Vimentin-positive SC arm indicates the sum of all cytoplasmic Vimentin signals reaching from the basal membrane up to the tubular lumen in Vimentin-stained testicular sections. (5–10 separate areas at 200×; *n* = 3 mice per genotype). Data are presented as mean ± SEM. **P < 0.01 by unpaired Student’s *t-*test. **D** Representative images of the F-actin filaments and anti-WT1 co-fluorescence staining in primary SCs from control and *Uhrf1* cKO mice at P21. Scale bar = 10μm. **E** The appearance of biotin tracer dye in the adluminal compartment of seminiferous tubules of adult *Uhrf1* cKO mice suggests a leaky BTB, while control mice show restriction of biotin tracer to the basal compartment. Scale bar=100μm. **F** Histogram showing the quantification of fluorescence distribution of Biotin in **E**. ***P < 0.001 by unpaired Student’s *t-*test. Each bar in the histogram presents a mean ± SEM of *n* = 3 mice, and 50 randomly selected round tubules per mouse were scored. **G**–**L** Immunofluorescent staining of basal ES proteins N-cadherin (**G**), β-catenin (**I**), tight junction protein ZO-2 (**K**) in control testes and *Uhrf1* cKO testes are shown, and histograms summarized results of fluorescence images regarding changes in the relative distribution of N-cadherin (**H**), β-catenin (**J**), ZO-2 (**L**) in *Uhrf1* cKO vs. control groups. Each bar in the histogram is a mean ± SEM of *n* = 4 mice, and 50 randomly selected round tubules were scored. Scale bar = 50μm. ***P < 0.001 by unpaired Student’s *t-*test. **M**, **N** Transmission electron microscopy (TEM) images of control (**M**) and *Uhrf1* cKO (**N**) testis at P56 are shown. *Uhrf1* cKO SCs had abnormally thin cytoplasmic arms, poor adherence to neighboring germ cell gaps, fragmented nucleoli, and massive accumulated lipids, while the control SC cytoplasmic arm was broad and tightly wrapped around germ cells. Abbreviation: SC Sertoli cell, Spg Spermatogonia, Spc Spermatocyte, RS Round spermatid, In indentation of the nuclear membrane, Nu nucleolus, Cc satellite chromocenters, Mt mitochondria, Lp Lipid.

Because the disruption of GC-SC adhesion was associated with cytoskeletal disorganization in SCs [31, 32], we performed staining of F-ACTIN, a marker for ectoplasmic specialization (ES) [33], to test whether the cytoskeletal structure was disorganized in UHRF1-deficient SCs. As shown in Fig. 4A, in control testes, F-ACTIN was prominently organized around the SC-spermatid interface (white arrowheads in Fig. 4A) and also at the SC-SC interface near the basement membrane (white arrows in Fig. 4A) to support the apical ES and basal ES function [34, 35]. However, in *Uhrf1* cKO testes, F-ACTIN no longer tightly localized at the basal ES to support the BTB but appeared as a truncated/branched network, diffusely localized at the adluminal compartment of seminiferous tubules (yellow arrows in Fig. 4A). In addition, immunostainings of *β*-ACTIN and *α*-TUBULIN verified disruption of organized actin and microtubular arrangement in *Uhrf1* cKO seminiferous tubules (Fig. 4B). Since Vimentin is a cytoskeleton protein in the cytoplasm of SCs, we next analyzed the localization of SC-intrinsic vimentin [36], which showed a shorter length of vimentin filaments of SCs in *Uhrf1* cKO seminiferous tubules compared with that of controls (Fig. 4B, C). Further examining the cytoskeletal structure in purified SCs by immunostaining with F-ACTIN revealed that F-ACTIN cytoskeleton in UHRF1-deficientSCs are disorganized compared with controls (Fig. 4D). Together, these results suggest that the ablation of UHRF1 in SCs disrupts the cytoskeletal organization of SCs, thereby compromising cell adhesion between GCs and SCs.

### UHRF1 specific deletion in SCs leads to the destruction of the BTB integrity

The cytoskeletal system in SCs is noted for its role in maintaining the structural and functional integrity of the BTB. We herein studied whether BTB integrity was compromised via biotin tracing assay in vivo. The results showed that the biotin tracer is able to permeate into seminiferous tubules in adult *Uhrf1* cKO testes at P56 (Fig. 4E, F). The BTB is also called the basal ES, which is a unique SC-SC structure composed of actin-based basal ES, tight junction (TJ), and gap junction [37]. As shown in Fig. 4G–L, in control testes, the basal ES proteins (N-cadherin and *β*-catenin) and TJ proteins (ZO-2) were tightly associated with the BTB (see white brackets in Fig. 4G, I, and K) located in the basement membrane in seminiferous tubules. However, in *Uhrf1* cKO testes, these proteins were found to be diffusely localized at the BTB by extending considerably away from the site (see yellow brackets in Fig. 4G, I, and K), well beyond the basement membrane, possibly due to disruptive changes in the organization of F-ACTIN. Furthermore, ultrastructural analysis by TEM revealed an intact BTB structure and clear SC nucleus morphology in control seminiferous tubules (Fig. 4M and Supplementary Fig. 5A). In contrast, abnormal alterations in BTB integrity with large irregular cavities and an accumulation of lipid droplets were observed in the seminiferous epithelium of *Uhrf1* cKO testes at P56 (Fig. 4N and Supplementary Fig. 5B), which suggests severe defects in the SC-SC and SC-GC junctions. In sum, these data indicate that UHRF1 in SCs is essential for maintaining the BTB integrity of SCs.

### Global gene expression changes in UHRF1-deficient SCs

**Fig. 5:**
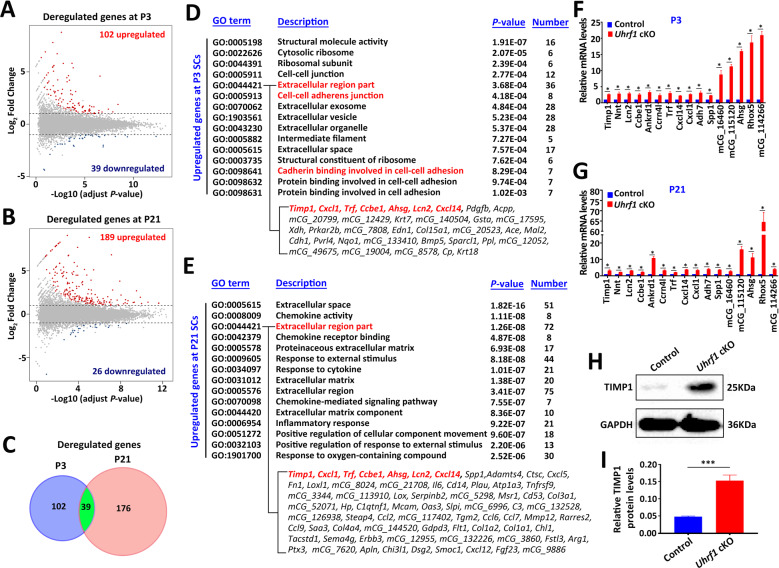
UHRF1 in SCs controls a transcriptional program of ECM and cell adhesion. **A**, **B** Volcano plots showing the RNA-seq data obtained from primary SCs of control and *Uhrf1* cKO mice at P3 (**A**) and P21 (**B**). Red dots indicate genes that were significantly upregulated, and dark blue dots indicate genes that were significantly downregulated (fold change>2, and FDR < 0.05) in *Uhrf1* cKO SCs. **C** The Venn diagram shows 39 common deregulated genes at two developmental time points, P3 and P21. **D**, **E** Gene ontology (GO) term analyses of the upregulated genes in *Uhrf1* cKO primary SC at P3 (**D**) and P21 (**E**) are shown. The top 15 enriched GO pathways in the upregulated genes of P3 are illustrated by gene counts and *P-*values. Genes related to extracellular region part and cell adhesion were significantly upregulated in P3 and P21 *Uhrf1* cKO SCs. **F**, **G** RT-qPCR analyses of 16 significant genes to validate RNA-Seq data from P3 (**F**) and P21 (**G**) primary SCs. Results are mean ± SEM. *n* = 3 mice. *P < 0.05 by unpaired Student’s *t-*test. **H** Western blot analysis showing TIMP1 protein in P21 primary SCs from control and *Uhrf1* cKO testes. GAPDH was used as a loading control. **I** Quantification of the expression of the protein shows the significantly elevated levels of TIMP1 in *Uhrf1* cKO groups. Data are presented as mean ± SEM, *n* = 3 mice. ***P < 0.001.

To gain insight into the molecular mechanism underlying UHRF1 regulation of SC development and the phenotypes of *Uhrf1* cKO mice, we purified SCs from both control and *Uhrf1* cKO testes at P3 (SCs in proliferation) and P21 (SCs in differentiation) to perform transcriptome profiling by RNA-sequencing. In total, 141 and 215 genes with >2-fold altered expression levels in *Uhrf1* cKO SCs at P3 and P21, respectively, were identified as differentially expressed genes (DEGs) compared to the control (Fig. 5A, B and Supplementary Tables 1, 2). Among these, 102 genes were upregulated, and 39 genes downregulated in *Uhrf1* cKO SCs at P3, whereas 189 genes were upregulated and 26 genes were downregulated in *Uhrf1* cKO SCs at P21 (Fig. 5A, B). Interestingly, 39 DEGs were common at P3 and P21 SCs (Fig. 5C), representing 27.7% and 18.1% of the DEGs in *Uhrf1* cKO SCs at P3 and P21, respectively. The gene ontology (GO) analysis discovered that the upregulated transcripts at P3 SCs are most significantly enriched for those that function in extracellular regions, the extracellular exosome, extracellular vesicles, and cell-extracellular organelles (Fig. 5D). Following the same criterion, we observed that genes significantly upregulated at P21 included those most closely associated with the extracellular region, extracellular space and response to external stimulus (Fig. 5E). To validate the RNA-seq findings, we selected a set of genes known to be implicated in ECM homeostasis and intracellular events to perform RT-qPCR in isolated primary SCs. In line with our sequencing results, further RT-qPCR in isolated primary SCs confirmed that the mRNA levels of a set of selected genes were all significantly increased in *Uhrf1* cKO SCs at both P3 and P21 in comparison to controls (Fig. 5F, G). Importantly, we found that the protein level of TIMP1 (a biomarker of ECM metabolism) was significantly elevated in *Uhrf1* cKO SCs at P21 compared to controls (Fig. 5H, I), suggesting the post-transcriptional regulation of the genes related to ECM metabolism was also affected upon loss of UHRF1 in SCs. Collectively, these analyses further suggest that UHRF1 in SCs might be responsible for regulating the expression of the ECM and cell adhesion genes at both transcriptional and post-transcriptional levels, which is critical for the functional development of SCs.

### UHRF1 regulates ECM and cell adhesion related genes in SCs by CG methylation maintenance

**Fig. 6:**
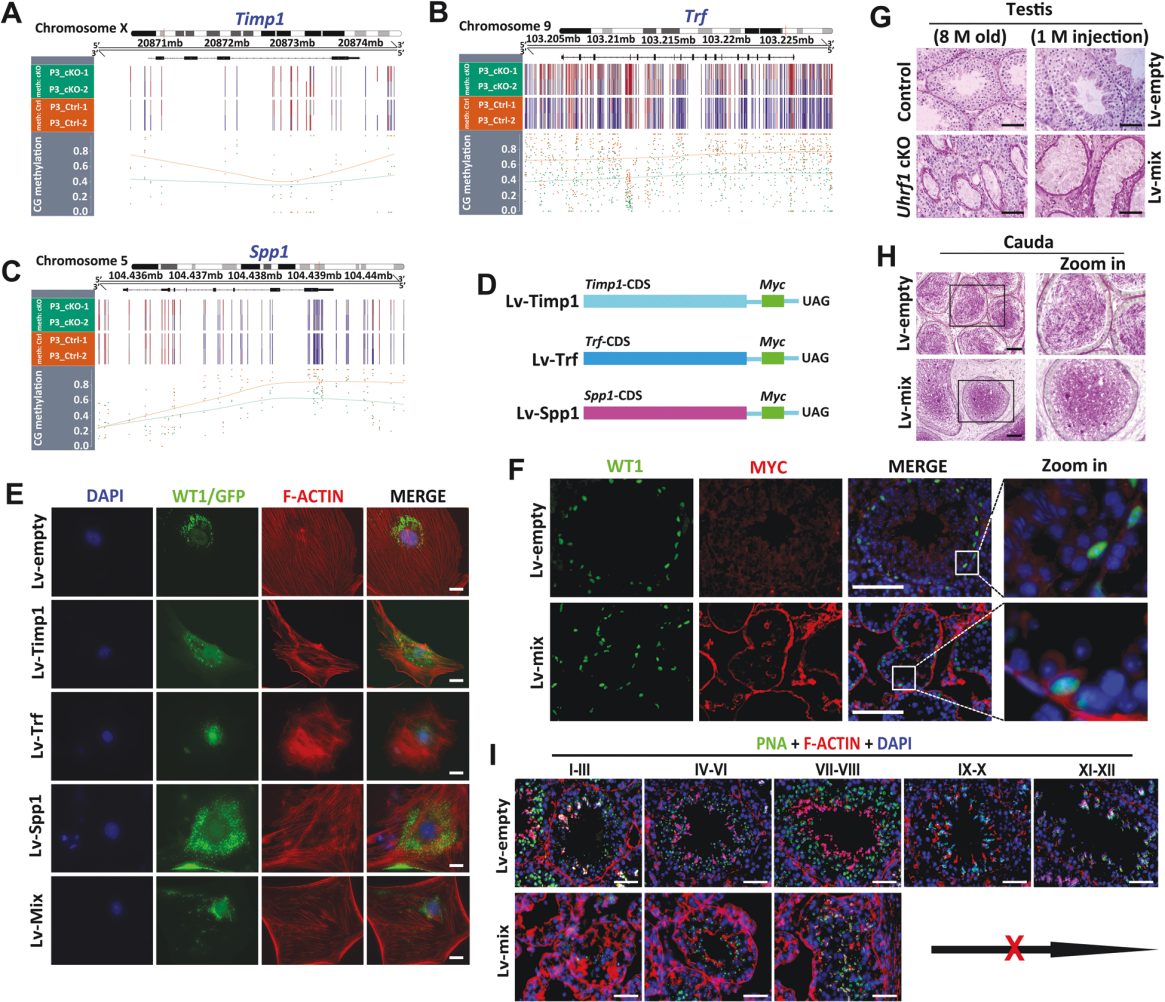
Overexpression of ECM- and cell adhesion-related genes (*Timp1, Trf*, and *Spp1*) results in a similar phenotype with *Uhrf1* cKO SCs and testes. **A–C** Methylation profile for the *Timp1* (**A**), *Trf* (**B**), and *Spp1* (**C**) locus across the genome. **D** Schematic representation of *Timp1, Trf*, and *Spp1* lentiviral overexpression vectors construction are shown. CDS: gene coding region; Myc: tag protein; UAG: termination codon. **E** Lentiviral supernatants of *Lv-Timp1*, *Lv-Trf*, *Lv-Spp1*, and three gene vectors mixture (Lv-mix), or Lv-empty alone were transfected into primary SCs of WT mice at P21. WT1 as a SC marker expressed in nuclear. GFP represents the vector expression. Scale bars = 10μm. **F** Co-immunostaining of WT1 and MYC to verify the overexpression in SCs after 10 days for injection of concentrated lentiviral supernatants of Lv-Timp1/Trf/Spp1 mixed vectors (Lv-mix), or Lv-empty. Scale bars = 100μm. **G** PAS staining showing the histology of testis sections from control and *Uhrf1* cKO mice at 8 months (*left*), and after 1 month of lentiviral supernatants injection (*right*). Scale bars = 100μm. **H** PAS staining showing the histology of cauda epididymis from Lv-empty and Lv-mix mice after 10 days of lentiviral supernatants injection. Scale bars = 100μm. **I** Co-immunofluorescence of PNA and F-ACTIN on testis sections from Lv-empty and Lv-mix mice after 10 days of lentiviral supernatants injection. Scale bars = 50μm.

Since our RNA-seq data showed that mRNA levels of ECM- and cell adhesion-related genes, including *Timp1, Trf, Spp1*, *Ccbe1*, *Lcn2*, and *Ahsg*, were significantly upregulated in *Uhrf1* cKO SCs, we asked whether these genes demethylated in *Uhrf1* cKO SCs. We thus performed WGBS and examined the CG methylation status of those genes across the genome. The results showed that *Timp1*, *Trf*, *Spp1*, *Lcn2, Ahsg*, and *Ccbe1* appeared to be demethylated in the genome (Fig. 6A–C and Supplementary Fig. 6A–C). In addition, the Kyoto Encyclopedia of Genes and Genomes (KEGG) analysis of WGBS revealed that genes with DNA hypomethylation in *Uhrf1* cKO SCs at P3 were most significantly enriched for cell focal adhesion (Supplementary Fig. 6D). Consistent with the WGBS data, bisulfite DNA sequencing revealed methylation levels in promoter regions of *Timp1*, *Trf*, and *Spp1* are reduced in *Uhrf1* cKO SCs at P3 and P21 compared to those of control SCs (Supplementary Fig. 7A–C). These results suggest that UHRF1 can regulate the expression of ECM- and cell adhesion-related genes in mouse SCs through CG methylation maintenance in the genome.

**Fig. 7:**
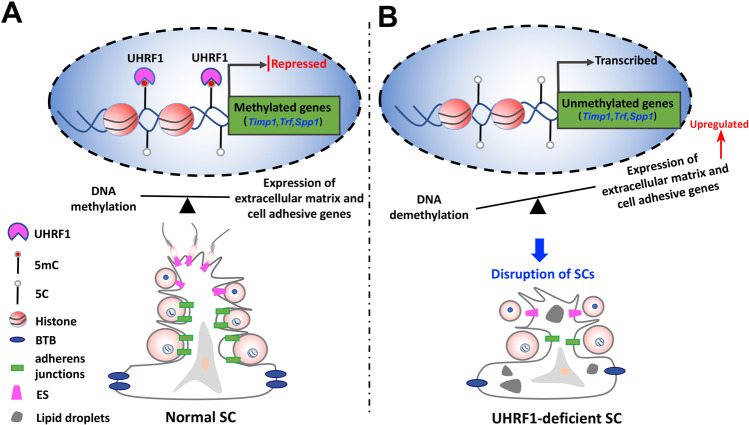
Working model for the functions of UHRF1 in SCs, as described in the discussion. **A** In normal conditions, UHRF1 in SCs controls a coordinated transcriptional program of ECM and cell adhesion genes (*Timp1, Trf*, and *Spp1*, et al.) in SCs by regulating its CG methylation, which usually plays a critical role in the coordinated homeostasis of adhesion between SC-GC and the cyclical disassembly versus reassembly of BTB. **B** In UHRF1-deficient SCs, the expressions of ECM and cell adhesion genes (*Timp1, Trf*, and *Spp1*) were upregulated due to their DNA demethylation, which leads to BTB disruption, junction defects, and inadequate cell adhesion, thereby resulting in a massive loss of immature germ cells from the seminiferous epithelium.

To test whether the above-identified UHRF1 regulated genes by DNA methylation indeed have a functional role in the SCs structure and GC-SC adhesion process, we chose the three most significantly upregulated genes (*Timp1*, *Trf*, and *Spp1*) that related with ECM and cell adhesion and overexpressed them in vitro (SCs) and in vivo (testes) using lentiviral infection (Lv). The results showed that the cytoskeletal structure of SCs in Lv-*Timp1*, Lv-*Trf*, Lv-*Spp1*, and Lv-*Timp1/Trf/Spp1* (Lv-mix) transfection groups were all disrupted, of which the cellular actin filaments (F-ACTIN) showed abnormal cross-alignment and accumulation compared with the Lv-empty control group (Fig. 6D, E). We then injected three gene mixtures with a lentiviral vector (Lv-mix) and an empty vector (Lv-empty) into the adult mouse seminiferous tubules to examine the functional role of overexpressing these genes in vivo*.* We found that MYC was detected in SCs in the Lv-mix group after 10 days (Fig. 6F), suggesting successful overexpression of *Timp1*, *Trf*, and *Spp1* in SCs in vivo. After one month of the lentivirus injection, most seminiferous tubules displayed empty in the Lv-mix mouse testes, which is phenocopied the *Uhrf1* cKO testes at eight months (Fig. 6G). Meanwhile, many immature GCs were observed in the cauda epididymis in Lv-mix injection mice, like *Uhrf1* cKO mice (Figs. 2D and 6H). In addition, we also observed a decrease in the number of elongated spermatozoa and abnormal F-ACTIN accumulation in Lv-mix injected mouse testes (Fig. 6I). This phenotype was akin to that of *Uhrf1* cKO mouse testes (Fig. 4A), suggesting overexpression of *Timp1, Trf*, and *Spp1* may lead to abnormal spermatogenesis. Taken together, these data indicate that the loss of UHRF1 in SCs leads to hypomethylation of genes (e.g., *Timp1, Trf, Spp1*) involved in theECM and cell adhesion, which subsequently affects expression in SCs on molecular levels, thereby disrupting SC development and cytoskeletal organization (Fig. 7A, B).

## Discussion

Like the dynamic expression pattern of UHRF1 in different types of male GCs [24], UHRF1 also displayed a differential spatiotemporal expression pattern in the different developmental status of SCs in both humans and mice. In human fetal testis, we can detect UHRF1 expression in SCs but not in adult human testis. The same is true in mice, suggesting UHRF1 is mainly expressed in immature but not in mature SCs. Consistent with our observations, recent studies have demonstrated that UHRF1 tends to be highly expressed in proliferating cells but downregulated during differentiation or in a quiescent state of various cell types [38–40]. Thus, it is likely that UHRF1 functions in SC proliferation and differentiation. However, it is worth noting that SC proliferation reduction might not be the primary defect contributing to male sterility in our case, because *Insr* and/or *Igf1r* mutants did not exhibit impaired reproductive ability despite the reduced proliferation rate of immature SCs during the late fetal and early neonatal testicular period [41].

In fact, in this study, we found that a large proportion of SC nuclei in the *Uhrf1* cKO testis are not located along the basal membrane as in the control testis from P14 to P56, indicative of disrupted SC polarity. This may give rise to male infertility because adult SCs need to be polarized so they can simultaneously support earlier-stage spermatogenic cells basally and later-stage cells apically [42]. A previous study showed that conditional knockout *Ptbp2* in GCs results in disorganization of F-ACTIN filaments in SCs, loss of SCs polarity, and destruction of SC-GC communication, which eventually leads to increased apoptosis and premature release into the lumen [43]. Likewise, in *Uhrf1* cKO cauda epididymis, we observed an excess of apoptotic and immature sperm, which further supports the notion that the primary *Uhrf1* cKO defect is a disrupted adhesive function of the SCs and their adhesion to GCs.

DNA methylation serves an essential role in connecting the cytoskeleton and ECM mechanics. The ECM in vivo is of particular importance for cellular fate-determination processes like cell development, migration, and maturation. Additionally, Landfors et al. suggested that dynamic DNA methylation in SCs coordinates with gene expression required during different stages of cell development and cell organization [44]. Herein, our results showed that UHRF1, mainly expressed in immature SCs, likely regulates genes that are essential for ECM-related processes and cell-to-cell interactions through its impact on DNA methylation. In line with the functional analyses, the RNA-Seq data further revealed that a set of genes including peptidase and protease inhibitors, cadherin, secreted phosphoprotein and claudin are differentially expressed upon lost function of UHRF1 in SCs. For instance, *Timp1* was mainly expressed in SCs and GCs, which was dynamically expressed during SC-SC and SC-GC junction assembly in vitro [45, 46], essential to counterbalance TNF-mediated activation of MMP-9 matrix metalloprotease, leading to collagen cleavage that perturbs SC tight junctions [47]. Consistently, in the present study, we found that both *Timp1* mRNA and protein levels increased in *Uhrf1* cKO SCs and that CG methylation status of *Timp1* hypomethylated in SCs, further suggesting that UHRF1 is the epigenetic regulator that physiologically orchestrates the methylation of genes participating in GC adhesion onto SCs within the seminiferous epithelium. Therefore, deregulation and demethylation of ECM-related and adhesion-associated genes, like *Timp1, Trf*, and *Spp1*, may directly affect *Uhrf1* depletion in SCs, leading to an imbalance between cell detaching proteolysis and cell-reattaching restructuring events that serve as the molecular basis for the observed testicular phenotype in *Uhrf1* cKO mice. This notion was further supported by overexpression of *Timp1, Trf*, and *Spp1* in SCs (in vitro) and testes (in vivo), which caused similar phenotypes with *Uhrf1* cKO mouse SCs and testes. This underlying molecular link between UHRF1 and its target genes could explain the abnormal morphology and physiology of SCs that induces the failure of developing GCs to firmly attach to nursing SCs and slough from the seminiferous epithelium of testis in postnatal day. However, we could not rule out the possibility of the alterations of transcriptional state and DNA methylation in fetal SCs due to the UHRF1 loss before birth and other signaling pathways essential for SC differentiation, as these had already been altered in fetal SCs.

In summary, our study demonstrates that exclusive expression of UHRF1 at early stages in the nuclei of SC lineage has long-reaching consequences into later stages, including control of ECM- and cell adhesion-related genes and repetitive elements directly in SCs via UHRF1-dependent CG methylation. The study conducted herein provided the first evidence that UHRF1-mediated DNA methylation in SCs is required for the normal cytoskeletal and extracellular architecture of SCs in mice. A further intriguing concept in this study revealed that UHRF1, as a maker of immature SCs, regulates SC function during spermatogenesis in humans and mice.

## Materials and methods

### Mice

All animal experiments were performed following the guidelines of the Institutional Animal Care and Use Committee (IACUC) of Tongji Medical College, Huazhong University of Science and Technology. All mice were housed in a specific pathogen-free facility under climate-controlled conditions with a 12-h light/dark cycle and were provided with water and a standard diet. Floxed *Uhrf1* mice (*Uhrf1*^*flox/flox*^) were obtained from the Shanghai Research Center for Model Organisms. The details were referred to in our previous article [24]. *Amh*-Cre mice in the C57BL/6 J background were purchased from the Jackson Laboratory. *Amh*-Cre mice were crossed with *Uhrf1*^*flox/flox*^ mice to generate the *Amh*-Cre; *Uhrf1*^*+/flox*^ females, then the *Amh*-Cre; *Uhrf1*^*+/flox*^ female mice were bred with *Uhrf1*^*flox/flox*^ male to obtain the *Amh*-Cre; *Uhrf1*^*flox/flox*^ (designed as *Uhrf1* cKO) males. Primers used for genotyping are listed in Supplementary Table 3.

### Human testicular tissue collection

Fetal testicular tissues were collected from pregnant women after an inevitable abortion, such as pre-mature rupture of membranes. Normal testicular tissue samples from the adult were collected from the remaining samples after microdissection of testicular sperm extraction (micro-TESE). All procedures were performed following the protocols approved by the Medical Ethics Committee of the Reproductive Medicine Center of Tongji Medical College of Huazhong University of Science and Technology. All participant patients have signed the informed written consent to the research process.

### SCs isolation and culture

For SCs isolation, the testes were removed and decapsulated in the DHANKS medium. Testicular seminiferous tubules were placed in a 15 ml centrifuge tube containing 1 mg/mL collagenase IV, 0.5 mg/mL Deoxyribonuclease I, and 0.5 mg/mL hyaluronidase in 10 ml DMEM/F12 medium for 10 min at 37 °C in a constant shaker. After removing the supernatant containing Leydig/interstitial cells by centrifugation (1000 rpm/min) for 10 min, the remaining seminiferous tubules were washed 3 times with DHANKS medium (10 min/wash). The seminiferous tubules were then incubated with 2.5 mg/ml trypsin and 0.5 mg/mL Deoxyribonuclease I solution for 10 min at 37 °C with shaking. The mixture containing SCs was filtered through 40μm pore-size nylon mesh, then washed with DHANKS medium and centrifuged at 1000 rpm three times for 5 min each. The pellet was suspended with complete DMEM/F12 medium without FBS and cultured in 24-well plates at 35 °C for 48 hours. After culture, cells were incubated with hypotonic shock solution (20 mM Tris-HCl, PH = 7.4) for 3 min, followed by DHANKS washing three times to remove male germ and other types of cells. Pure SCs were cultured for 24 hours and prepared for the experiment. Freshly isolated SCs were identified using immunostaining with antibodies against WT1 and achieved more than 95% cell purity and viability.

### Periodic acid-Schiff (PAS) staining and immunohistochemistry (IHC)

Mouse testes and epididymides were harvested from different time points and fixed in Bouin’s solution (Sigma, HT10132) at 4 °C overnight. Washed by 75% alcohol several times, the tissues were then embedded in paraffin. Following dewaxing and rehydration, 5μm sections were stained with periodic acid-Schiff (PAS) and imaged under the light microscope. After dewaxing and hydration, sections were microwaved for antigen retrieval by 0.01% Tris-EDTA (PH = 9.0) and cooled at room temperature (RT). Then sections were washed with PBS and treated with 3% H_2_O_2_ at RT for 15 min. After washing with PBS three times, the sections were blocked in 5% BSA for 1 h and then were incubated with primary antibody at 4 °C wet box overnight. The sections were washed with PBS and incubated with secondary antibody for 1 h at RT. After washing with PBS and colored with DAB at RT, the sections were stained with hematoxylin and washed with ddH_2_O. After hydration, sections were mounted with neutral resin and then photographed with a white light microscope.

### Immunofluorescence

Testes were fixed with 4% paraformaldehyde (PFA) diluted with PBS and then were gradient dehydrated with 5, 10, 12.5, 15, and 20% sucrose in PBS. After embedded in 50% Tissue-Tek O.C.T. compound (Sakura Finetek, 4583) in 20% sucrose on liquid nitrogen, the 5μm cryosections were spread onto Superfrost Plus slides and stored in −80 °C. After antigen retrieval by 0.01 mM Citrate or Tris-EDTA (PH = 9.0) and washing three times in PBS (10 min/wash), sections were incubated with primary antibodies (Supplementary Table 4) in a humidified chamber at 4 °C overnight. The slides were washed three times with PBS and then incubated with a secondary antibody (Supplementary Table 4) at RT for 1 h. After the primary SC was cultured, the sections were permeabilized with 0.2% Triton X-100 at RT for 5 min and washed with PBS. Then the sections were incubated with primary and secondary antibodies, mounted with DAPI (H1200, Vector laboratories), and photographed. For F-ACTIN staining, cryosections were rinsed with PBS and permeabilized by 10% Triton X-100 at RT for 30 min. Then the sections were incubated with phalloidin (Molecular Probes) and Lectin PNA (Molecular Probes) at RT for 1 h. After washing three times with PBS (10 min/wash), the slides were counterstained with DAPI and photographed with a fluorescence microscope (Olympus, Japan).

### EDU staining

The EDU staining was referred to as the protocol of the BeyoClick™ EdU-488 kit. Briefly, primary SCs were cultured with 10 nm EDU for 24 h and fixed with 4% PFA for 15 min at RT. Then sections were permeabilized with 0.3% Triton X-100 for 10 min. After washing with PBS, sections were incubated with Click reaction solution for 1 h, then washed with PBS and mounted with DAPI for the photograph.

### Western blotting

SCs were collected and washed by PBS and lysed by RIPA buffer (CWBIO, Cat# 01408). Samples were heated at 100 °C for 10 min in protein lysis buffer (50% RIPA lysis buffer and 50% protein loading buffer) and stored at −20 °C until use. In total, equal protein lysates were separated on a 12% SDS-PAGE gel and transferred to PVDF membranes (Bio-red, America). The membranes were blocked for 1 h with 5% non-fat milk at RT and then incubated with primary antibodies at 4 °C overnight. After three washes in TBST (10 min/wash), the membranes were incubated with the HRP-conjugated secondary antibody for 1 h at RT. ECL (ClarityTM Western ECL Substrate, Bio-Rad) was used for chemiluminescence detection and membranes were photographed by ChemiDoc XRS + system (BIO-RAD). The band intensity was quantified by densitometry using Image J Analysis software (Research Services Branch). Antibodies are listed in Supplementary Table 4.

### Transmission electron microscopy (TEM)

TEM was performed as our previous study described with minor modifications [48]. Briefly, mouse testes were fixed in 4% PFA containing 3% glutaraldehyde and staining with 1% uranyl acetate for 2 h at 4 °C, then for 1 h at RT. After three washes with 0.1 M cacodylate buffer, tissue was dehydrated and embedded in Eponate mixture (Electron Microscopy Sciences, Hatfield, PA, USA) for polymerization about 24 h at 60 °C. 70 nm sections were cut and counterstained with methanolic uranyl acetate and lead citrate. Sections were photographed using a transmission electron microscope (CM120 BioTwin Philips).

### TUNEL staining

Testes were fixed in 4% PFA in PBS, and then were gradient dehydrated and embedded in liquid nitrogen. 5μm cryosections were cut and stored at −80 °C. TUNEL staining was performed using One Step TUNEL Apoptosis Assay Kit (Green) (Beyotime, C1086) according to the manufacturer’s instructions. Images were obtained with an immunofluorescence microscope (Olympus, Japan).

### Flow cytometry

The testicular cells from P21 mice being digested were collected and incubated with 70% ice-cold methanol for 30 min. Followed by treatment with PBS including RNase, propidium iodide, and 0.2% Triton X-100, the cells were analyzed using a flow cytometer (BD).

### BTB integrity assay in vivo

Mice were anesthetized using pentobarbital sodium (30–50 mg/kg). Testes were exposed by a small incision through the scrotal layer. A small opening in the tunica albuginea was gently created with fine forceps and 50 μl of 10 mg/ml fluorescein isothiocyanate (FITC)-conjugated inulin (F3272; Sigma) was injected using a Hamilton syringe into the interstitium of one testis of each animal. After 30 min of injection, the animals were euthanized by cervical dislocation, and the testes were immediately removed and fixed in 4% PFA solution at 4 °C for overnight. Then the testes were dehydrated with 5, 10, 12.5, 15, and 20% sucrose for 30 min respectively. The tissues were embedded and cut for staining. Sections (5μm) were incubated with Alexa Fluor 488 streptavidin (1:500; Thermo Fisher Scientific), and slides were treated with DAPI counterstain for fluorescence imaging. BTB integrity quantitation analyses were performed as in the previous report [49]. Briefly, the ratio of the distance of the FITC-conjugated streptavidin (D_Biotin_) from the basement membrane in a seminiferous tubule to the radius of a seminiferous (D_Radius_) was calculated in each tubule for quantification of BTB integrity. For oblique sections of the tubules, D_Radius_ was obtained using the average of the shortest and the longest distance from the basement membrane. About 50 tubules were randomly selected from each testis of *n* = 3 for control and *Uhrf1* cKO mice.

### RNA extraction and RT-PCR

Total RNA was isolated from SCs using Trizol reagent (Life Technologies) following the manufacturer’s procedure. RNA concentration and purity were assessed using the Nanodrop ND-2000 spectrophotometer (Thermo Scientific). A total of 500-1000 ng RNA was reverse transcribed according to the High-Capacity cDNA Reverse Transcription Kit to obtain complementary DNAs (cDNAs) (Thermo Scientific). RT-qPCR was performed with SYBR green master mix (TSINGKE) on the LightCycler@96 Real-Time PCR system (Roche) according to manufactures’ instructions. The specific primers are listed in Supplementary Table 3.

### Genomic DNA isolation and bisulfite sequencing

SCs were isolated from testes and were lysed with the lysis buffer following the manufacturer’s instructions of TIANamp Genomic DNA Kit. The bisulfite conversion of the genomic DNA (2 μg) was performed by the manufacturer’s protocol of the EpiTect Bisulfite Kit (QIAGEN). Bisulfite-treated DNA was used to amplify by PCR amplification and was performed with an EpiTaq HS (TaKaRa) under the following conditions: denaturation at 98 °C for the 60 s and 35 cycles each of 98 °C for 10 s, annealed temperature (depends on the gene) for 30 s, and 72 °C for 45 s. The amplified regions were cloned into pClone007 (TSINGKE), sequenced, and then analyzed with QUMA (http://quma.cdb.riken.jp/top/quma_main_j.html). The primer sequences for PCR amplification are listed in Supplementary Table 3.

### RNA-Seq analysis

1 μg total RNAs from P3 and P21 mouse SCs (two biological repeats for control and *Uhrf1* cKO, respectively) were used for stranded RNA sequencing library preparation using KC-DigitalTM Stranded mRNA Library Prep Kit for Illumina® (Catalog NO. DR08502, Wuhan Seqhealth Co., Ltd. China) following the manufacturer’s instruction. After filtering the original sequencing data with Trimomatic (version 0.36), sequence alignments were performed to ensure that the homology is greater than 95%. After eliminating all errors and deviations caused by PCR amplification and sequencing, the EDGER package (version 3.12.1) was used to annotate and identify differentially expressed genes among different groups based on the UCSCMM10 mouse genome. The FDR corrected *P*-value cut off of 0.05 and fold-change cut-off of 2 was used to judge the statistical significance of gene expression differences. Gene ontology (GO) analysis and genomes (KEGG) enrichment analysis for differentially expressed genes were both performed by KOBAS software (version: 2.1.1) with a corrected *P*-value cut off of 0.05 to judge statistically significant enrichment.

### Whole-Genome Bisulfite Sequencing (WGBS) library preparation and sequencing and analysis

For WGBS library preparation, genomic DNA of SCs from 2-3 P3 mice of each phenotype were lysed with the lysis buffer following the manufacturer’s instructions of the TIANamp Genomic DNA Kit. WGBS library preparation was performed as described previously [50] with some modifications. In brief, after adding N6-methyladenine-free Lambda DNA as a spike-in control to assess bisulfite conversion efficiency, genomic DNA (100 ng) from SCs was sheared to 150-750-bp with ultrasound. Then the sample DNA was repaired and A-tailed by and ligated with NEBNext methylated adapter oligos for Illumina (New England BioLabs). The end-repaired and A-tailed DNA samples were then bisulfite converted with EZ DNA Methylation-Gold kit (Zymo Research) according to the manufacturer’s instructions. After bisulfite conversion, the converted DNA fragments were amplified for seven cycles with KAPA HiFi HotStart Uracil+ Readymix (Roche). After being cleaned with Agencourt AMPure XP beads (Beckman Coulter), the purified WGBS libraries were then sequenced on an Illumina HiSeq 1500. For analysis, the raw reads were performed to remove residual adapter sequence and reads with low-quality scores with SOAPnuke (version - 2.0.5). Clean reads were then mapped to sequence reads to the Mouse Genome Overview GRCm38 assembly (http://ftp.ensembl.org/pub/release87/fasta/mus_musculus/dna/), the reference genome using Bismark (version 0.20.1). The depth and coverage of chromosomes were calculated with samtools (version- 1.4) and bedtools (version- 2.26.0). Methylation detection was performed by Bismark, which calculates the proportion of methylated reads at every genome site. The differentially methylated regions (DMRs) between different groups were detected with metilene (version-0.2-7). The pathway enrichment analyses of DMR annotated genes were conducted with KOBAS (version 2.1.1).

### Preparation of *Timp1*, *Trf*, and *Spp1* overexpression construct

Total RNA was isolated from the adult testis and then was carried out to prepare cDNA by reverse transcription. The target genes of the full-length open reading frame were amplified from cDNA by PCR using specific primers. The PCR product was cloned to pCDH-CMV-MCS-EF1-CopGFP-T2A-Puro (MiaoLing Plasmid Sharing Platform). The recombinant vector pCDH-GFP-Timp1/Trf/Spp1 was named LV-Timp1, Lv-Trf, and Lv-Spp1, respectively. The empty vector pCDH-GFP-empty (named LV-empty) was used as a negative control. With the packaging plasmids (PSPA and PMDIG), high-quality proviral DNA was transfected to 293 T cells. After 48-72 hours, the lentivirus propagation of target genes was collected and concentrated for in vitro transfection into SCs and in vivo injection into testes.

### Lentiviral plasmids injection of testes in vivo

A high-titer virus of Lv-Timp1/Lv-Trf/Lv-Spp1 mixture or Lv-empty was delivered to mouse testes via injection into seminiferous tubules by a micropipette as described by Michaelis, M [51]. Crystal violet (0.02%) was added to the virus suspension to monitor the filling of the seminiferous tubules. Approximately 20 µl of viral suspension was injected into rete testis, filling more than 90% of the seminiferous tubules with the viral suspension. 10 and 30 days after injection, the testes were collected for IMF and PAS staining to assess the phenotype. Viral transfection in testes was verified by GFP or MYC detection.

### Lentiviral plasmids infection of SCs in vitro

For target genes overexpression, lentivirus was produced in HEK293T cells by separately co-transfecting with Lv- Lv-Timp1, Lv-Trf, and Lv-Spp1, or LV-empty. Then isolation of SCs and on day 1 of SCs cultures, either lentivirus empty alone or Lv-Timp1, Lv-Trf, and Lv-Spp1 alone or a mixture of three target genes was applied to the 24 wells. On day 3, all samples were harvested for immunofluorescence examination.

### Statistical analysis

Data were analyzed by Student *t*-test using SPSS 19.0, one-way ANOVA, and GraphPad Prism 7.0. The results were presented as mean ± SEM or SD, and significance was assumed for *P* < 0.05. Asterisks indicate: *P < 0.05; **P < 0.01; ***P < 0.001; *ns*, not significant.

## Supplementary information

Supplementary Figures

Reproducibility checklist

Supplementary Table 1

Supplementary Table 2

Supplementary Table 3

Supplementary Table 4

## Data Availability

All data needed to evaluate the conclusions in the paper are present in the article and/or the Supplementary Materials. All RNA sequencing data are deposited in the NCBI SRA (Sequence Read Achieve) database (PRJNA628503). All other supporting data of this study are available from the corresponding author upon reasonable request.
