# Supplementary material for: UHRF1 establishes crosstalk between somatic and germ cells in male reproduction
Source: Cell Death Dis. 2022 Apr 19;13(4):377. doi: 10.1038/s41419-022-04837-2 (PMC9018721; doi:10.1038/s41419-022-04837-2)

Supplementary Materials for

UHRF1 establishes crosstalk between somatic and germ cells in male reproduction

Yanqing Wu, Peng Duan, Yujiao Wen, Jin Zhang, Xiaoli Wang, Juan Dong, Qiang Zhao, Shenglei Feng, Chunyu Lv, Yang Guo, Satoshi H. Namekawa, Shuiqiao Yuan*

***Corresponding author Email:** shuiqiaoyuan@hust.edu.cn

This PDF file includes:

Supplementary Fig.1. Expression of UHRF1 in mature SCs cultured with or without 10% FBS.
Supplementary Fig.2. UHRF1 deficiency in SCs induces immature spermatid sloughing and seminiferous tubule atrophy.
Supplementary Fig.3. UHRF1 deficiency in SCs leads to germ cell apoptosis.
Supplementary Fig.4. UHRF1 deficiency in SCs leads to the number of SCs to decrease without apoptosis.
Supplementary Fig.5. Loss of UHRF1 in SCs results in structural cell-cell junction defects and lipid accumulation.
Supplementary Fig.6. Whole-Genome Bisulfite Sequencing (WGBS) revealed hypomethylation of genes in *Uhrf1* cKO SCs.
Supplementary Fig.7. Bisulfite sequencing showed the methylation levels in promoter regions of *Timp1*, *Trf,* and *Spp1* in control and *Uhrf1* cKO SCs at P3 and P21.

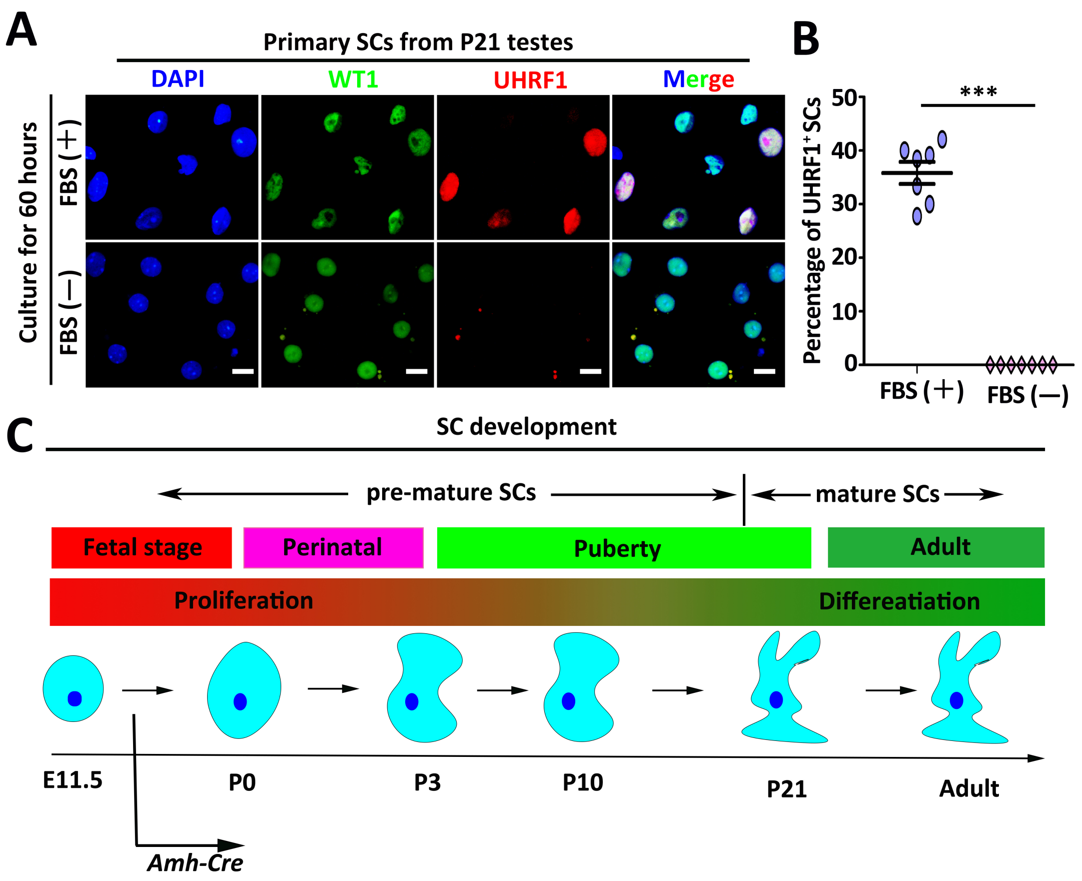

Supplementary Fig.1. Expression of UHRF1 in mature SCs cultured with or without 10% FBS. **(A)** Double immunostaining with UHRF1 and WT1 on P21 WT primary SCs cultured with 10% FBS and without 10% FBS for 24h is shown. Scale bar = 10μm. **(B)** Histogram showing the quantification of percent of UHRF1-positive SCs in (A). ***$P < 0.001$ by unpaired Student's *t*-test. **(C)** A schematic diagram shows SC development and the time point of *Amh*-Cre expression.

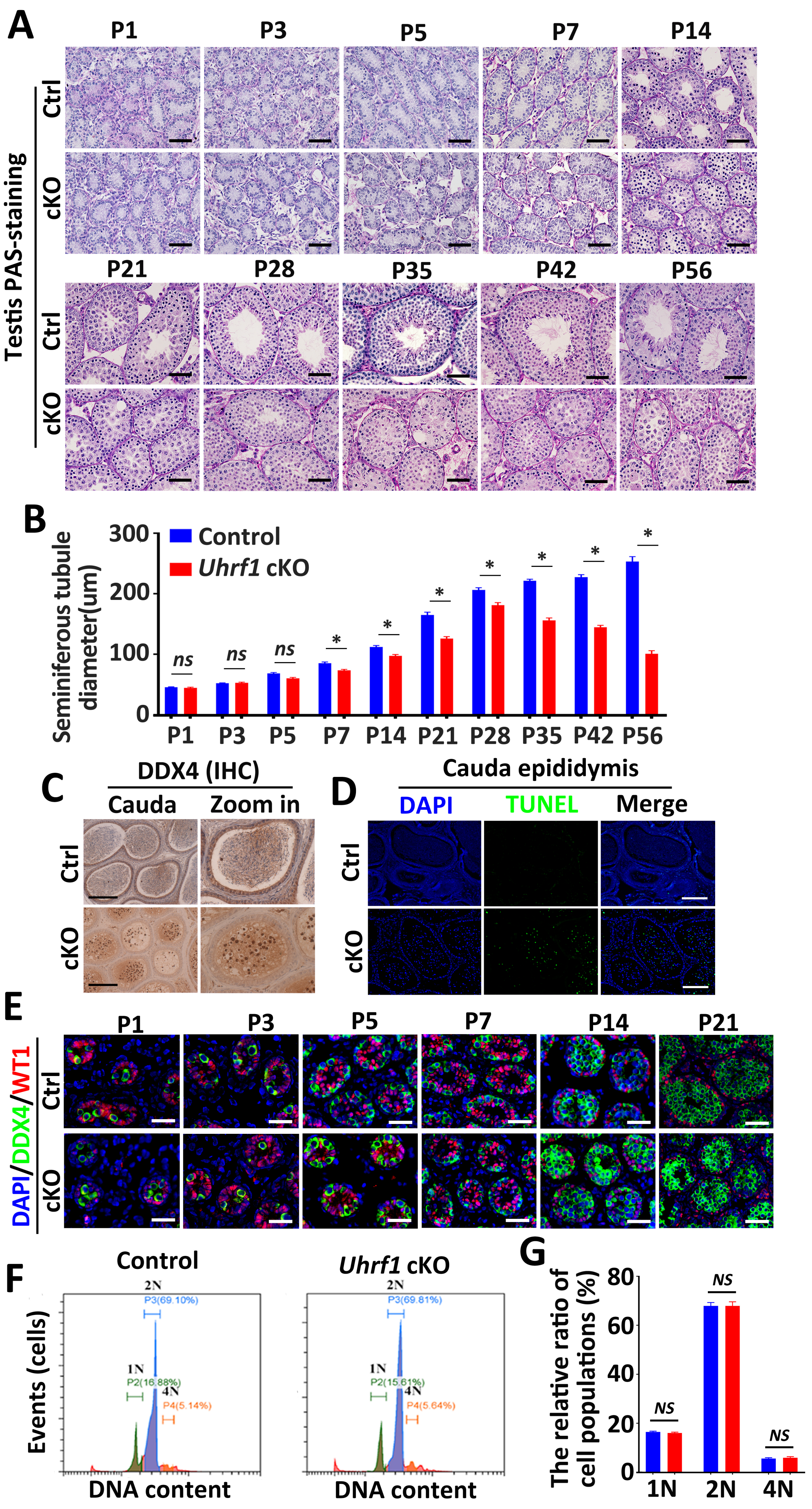

A
P1
P3
P5
P7
P14
Testis PAS-staining
Ctrl
cKO
P21
P28
P35
P42
P56
B
Seminiferous tubule diameter(um)
Control
Uhrf1 cKO
300
200
100
0
ns
*
P1 P3 P5 P7 P14 P21 P28 P35 P42 P56
C
DDX4 (IHC)
Cauda
Zoom in
D
Cauda epididymis
DAPI
TUNEL
Merge
E
DAPI/DDX4/WT1
P1 P3 P5 P7 P14 P21
F
Control
Uhrf1 cKO
2N
P3(69.10%)
P3(69.81%)
1N
P2(16.88%)
P2(15.61%)
4N
P4(5.14%)
P4(5.64%)
Events (cells)
DNA content
G
The relative ratio of cell populations (%)
80
60
40
20
0
NS
1N 2N 4N

Supplementary Fig.2. UHRF1 deficiency in SCs induces immature spermatid sloughing and seminiferous tubule atrophy. (A) Representative PAS-stained images of developing testes at P1, P3, P5, P7, P14, P21, P28, P35, P42, and P56 from control and *Uhrf1* cKO mice are shown. Scale bar = 50μm. **(B)** Quantification of seminiferous tubule diameter of testes of (b) is shown. n = 3 mice. Approximately 30 round seminiferous tubules per mouse were randomly chosen to count. *ns*, not significant, *P< 0.05 by unpaired Student's t-test. **(C)** Immunohistochemical staining of DDX4 is shown in P56 control and *Uhrf1* cKO epididymides. No DDX4-positive cell was observed in control, while many of DDX4^{+} cells can be detected in *Uhrf1* cKO cauda epididymis. Scale bars = 100μm. **(D)** Fluorescence of cauda epididymis from control and *Uhrf1* cKO mice is shown by TUNEL assay. Cells stained green are TUNEL-positive cells. Scale bars = 100μm. **(E)** Double immunofluorescent staining of DDX4 (Green) and WT1 (Red) is shown in control and *Uhrf1* cKO testes at P1, P3, P5, P7, P14, and P21. Scale bars = 50μm. **(F)** Flow cytograms show discrete peaks: a 1N (haploid) peak representing spermatids, a 2N (diploid) peak representing G1-phase spermatogonia and Sertoli cells, and a 4N (tetraploid) peak representing primary spermatocytes and G2-phase spermatogonia. **(G)** Relative amounts of three cell populations in the testes. Data are expressed as mean ± SEM of n = 3 mice. *ns*, no significant change by Student's t-test.

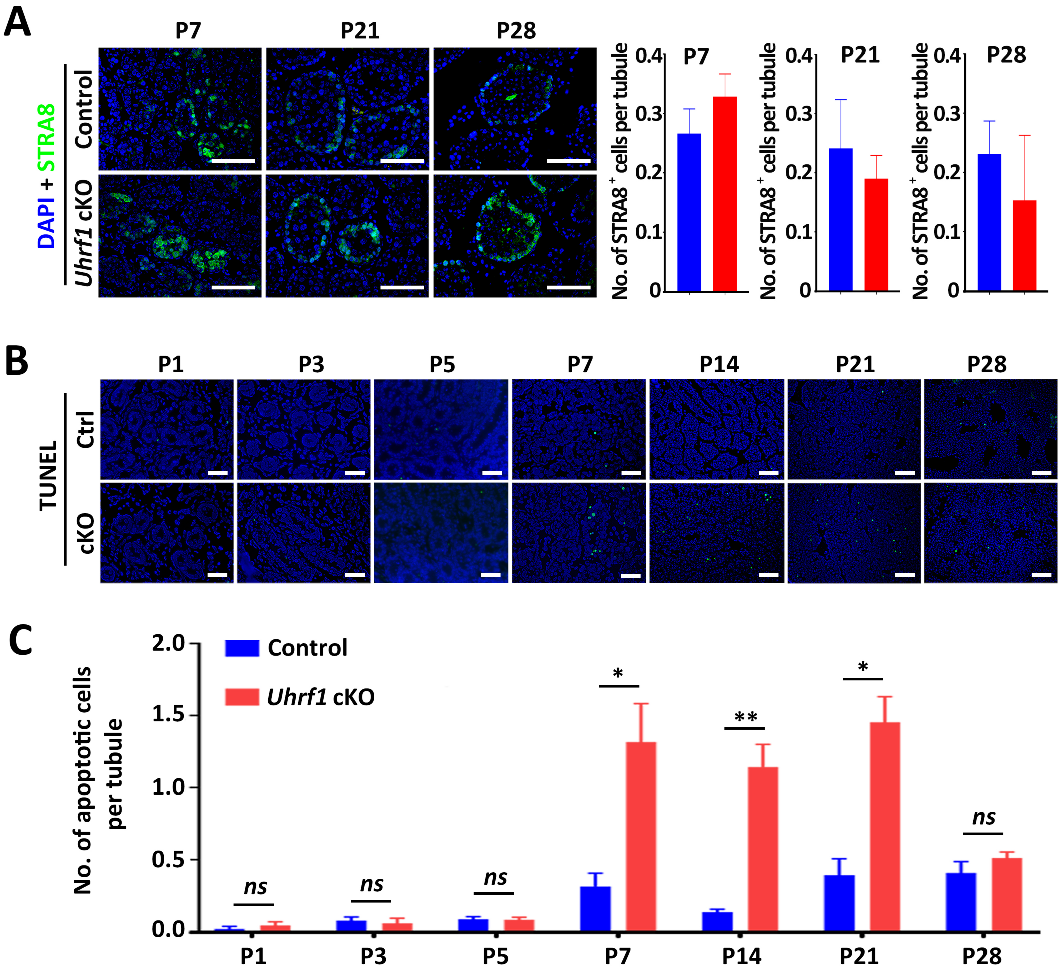

Supplementary Fig.3. UHRF1 deficiency in SCs leads to germ cell apoptosis. **(A)** Immunofluorescent staining for STRA8 on testis sections from control and *Uhrf1* cKO mice at P7, P21, and P28 are shown. Nuclei were stained with DAPI. Scale bar = 50μm. The right histograms show that the average number of $STRA8^+$ cells per tubule is no significant change in control and *Uhrf1* cKO testis. Results are mean ± SEM. **(B)** Representative result of TUNEL assays is showing the staining for frozen sections of control and *Uhrf1* cKO testes at P1, P3, P5, P7, P14, P21, and P28. Scale bars = 50μm. **(C)** The histogram shows the quantification of apoptotic cells (TUNEL positive cells) in (B). Data are presented as mean ± SEM, n = 6. *ns*, no significant change. **P*<0.05, ***P*<0.01 by unpaired Student's *t*-test.

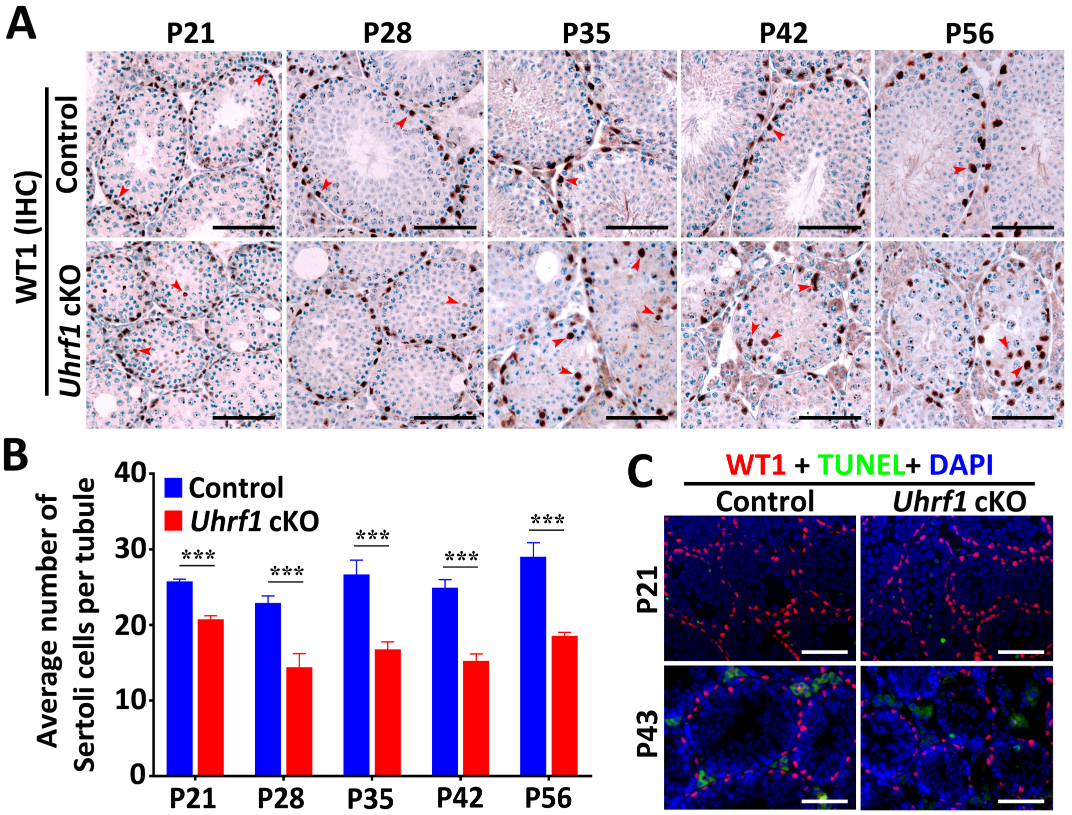

Supplementary Fig.4. UHRF1 deficiency in SCs leads to the number of SCs to decrease without apoptosis. **(A)** Immunohistochemical analysis of WT1 shows that numbers of SCs in *Uhrf1* cKO testes decreased at P21, P28, P35, P42, and P56 mice compared to control. Scale bar = 100μm. Red arrowheads indicate the nuclear of Sertoli cells. **(B)** The histogram shows the average number of $WT1^+$ cells per tubule reduced in *Uhrf1* cKO testis compared with control. Data are presented as mean ± SEM, n =3-7. ****P*<0.001 by unpaired Student's *t*-test. **(C)** TUNEL assay and WT1 co-staining on P21 and P43 testis frozen sections show no apoptosis SCs in *Uhrf1* cKO mice. Scale bar = 50μm.

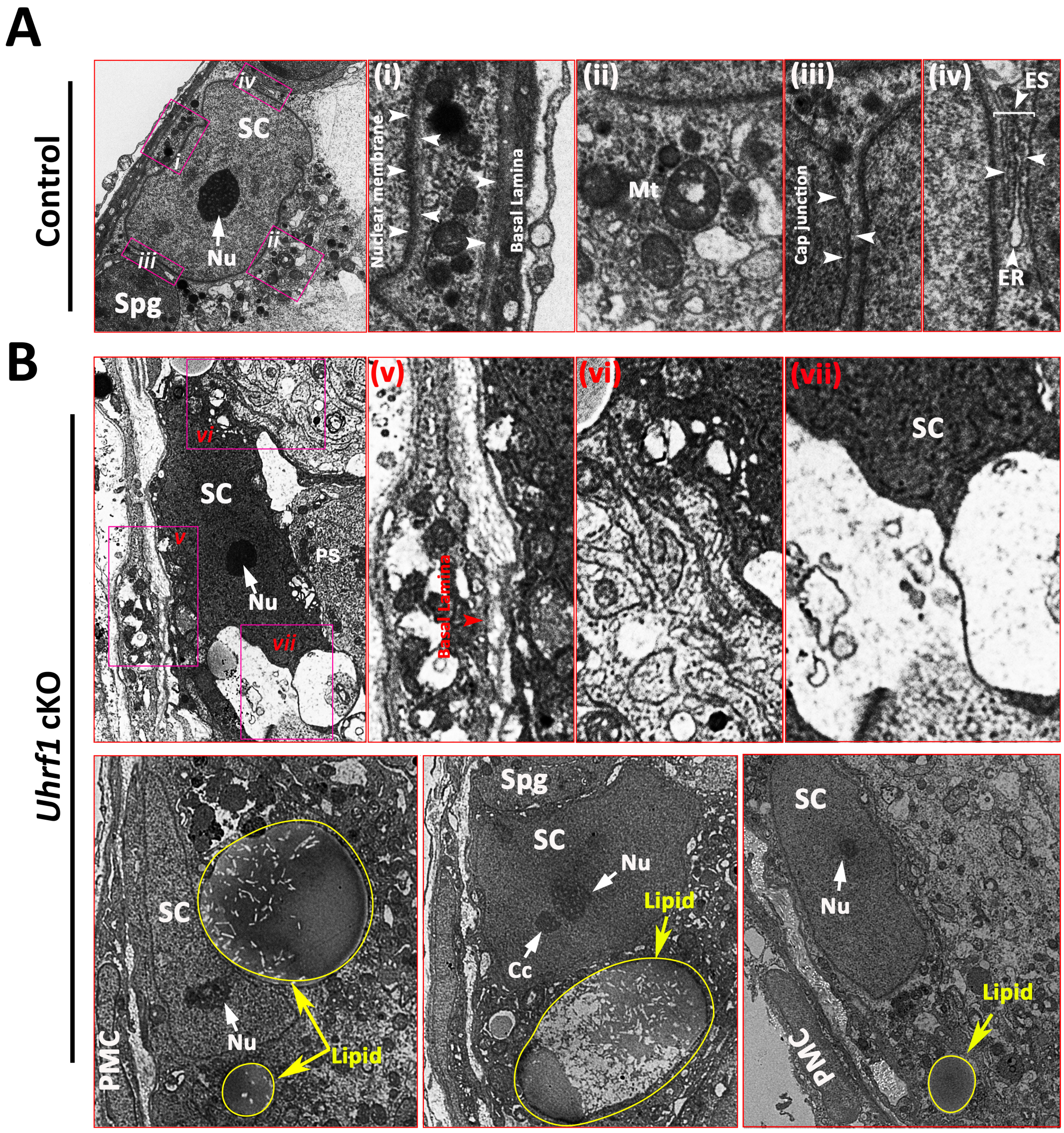

Supplementary Fig.5. Loss of UHRF1 in SCs results in structural cell-cell junction defects and lipid accumulation. (A) Transmission electron microscopy (TEM) images of control testis at P56 are shown. In control testes, BTB junctional complexes (i, ii, iii, iv) between Sertoli cells (SC) and germ cells were well-formed. **(B)** TEM images show that BTB complexes (v, vi, vii) of SCs were frequent discontinuities, poor adherence to neighboring germ cells, and lipid droplets (the yellow circle) were observed in SCs from *Uhrf1* cKO testis at P56. SC, Sertoli cell; Spg, Spermatogonia; Nu, nucleolus; Cc, satellite chromocenters; Mt, mitochondria; ER, endoplasmic reticulum; ES, ectoplasmic specialization; PMC, Peritubular myoid cell.

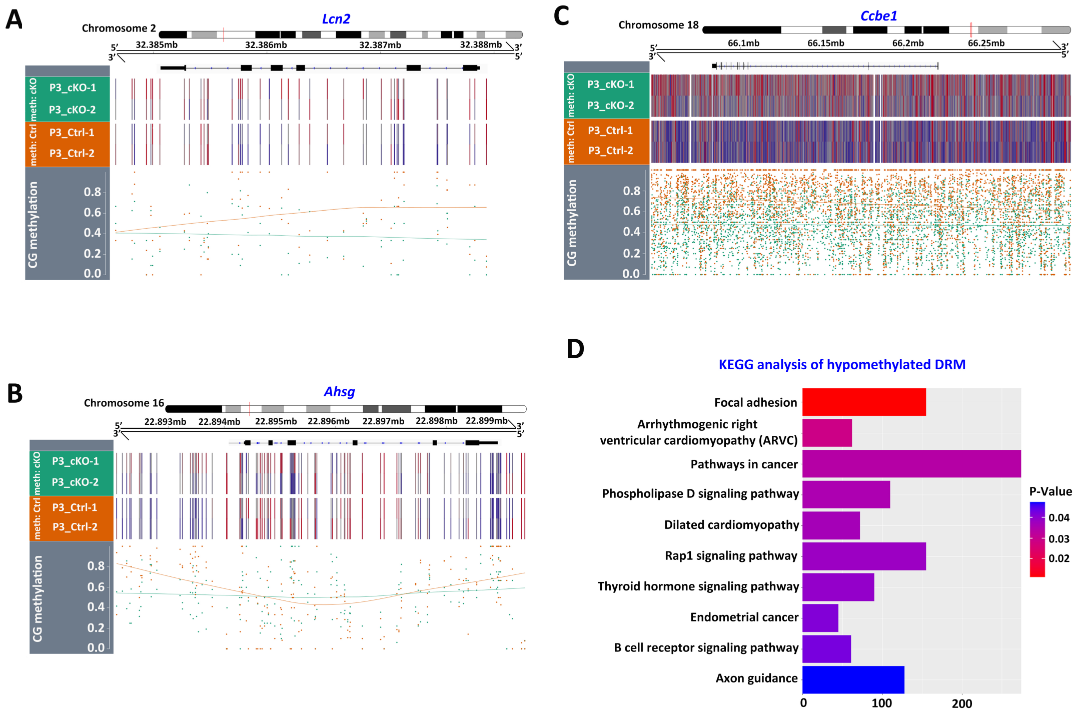

Supplementary Fig.6. Whole-Genome Bisulfite Sequencing (WGBS) revealed hypomethylation of genes in *Uhrf1* cKO SCs. **(A-C)** Methylation profile for the *Lcn2* (A), *Ahsg* (B), and *Ccbe1* (C) locus across the genome. **(D)** KEGG analyses of the CG methylation hypomethylated genes from control and *Uhrf1* cKO SCs at P3.

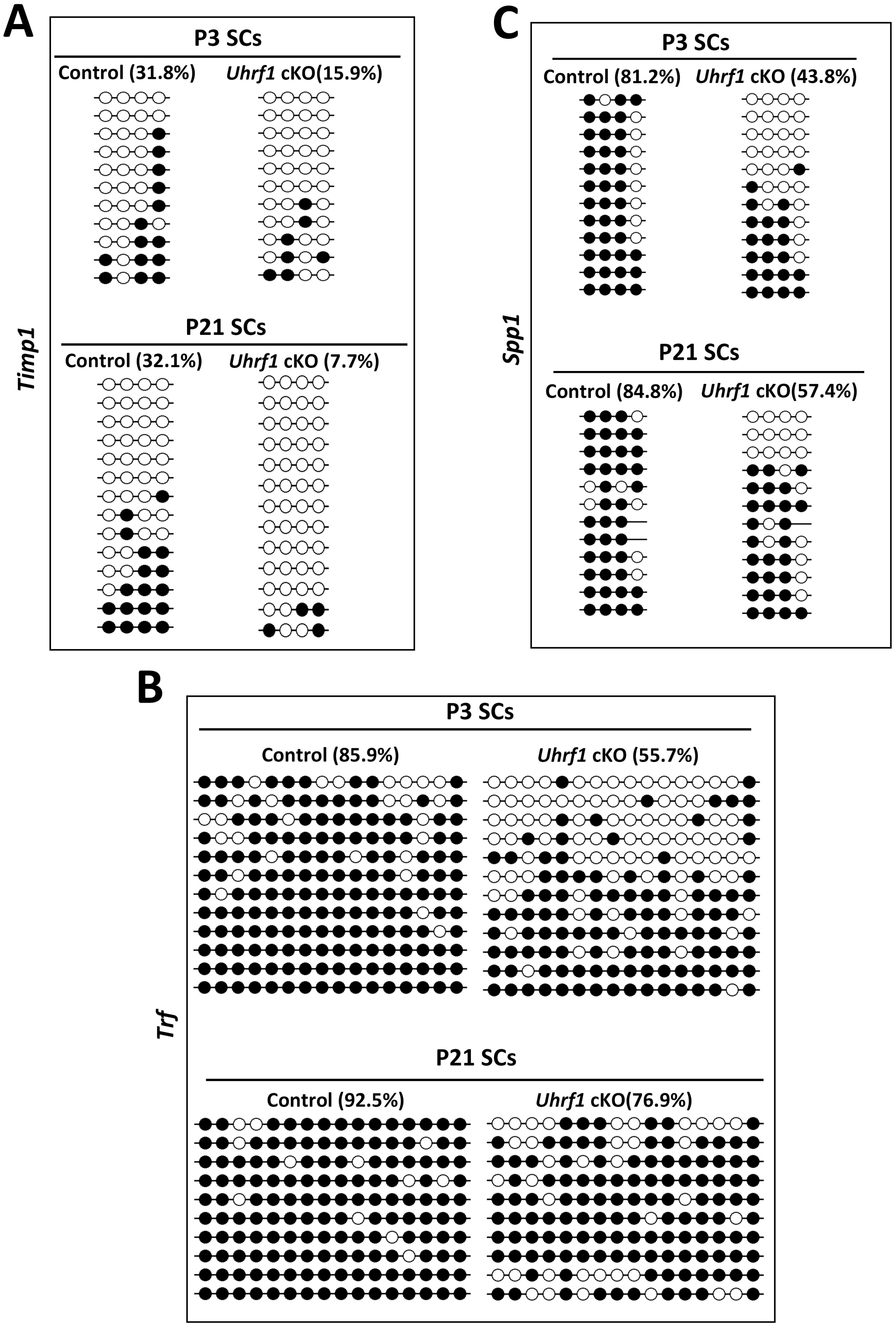

Supplementary Fig.7. Bisulfite sequencing showed the methylation levels in promoter regions of *Timp1*, *Trf,* and *Spp1* in control and *Uhrf1* cKO SCs at P3 and P21. (A-C) Bisulfite sequencing of CpG island of *Timp1* (A), *Trf* (B), and *Spp1* (C) in control and *Uhrf1* cKO SCs at P3 and P21 are shown, respectively. Methylated CpG sites are shown with black circles and unmethylated sites with open circles. The percentages of overall methylated CpGs are indicated.

Original images of Western blots related to Fig.5H

The upper panel was incubated with GAPDH antibody (Proteintech), and the lower panel was incubated with TIMP1 (R&D Systems). We showed the red rectangular area.

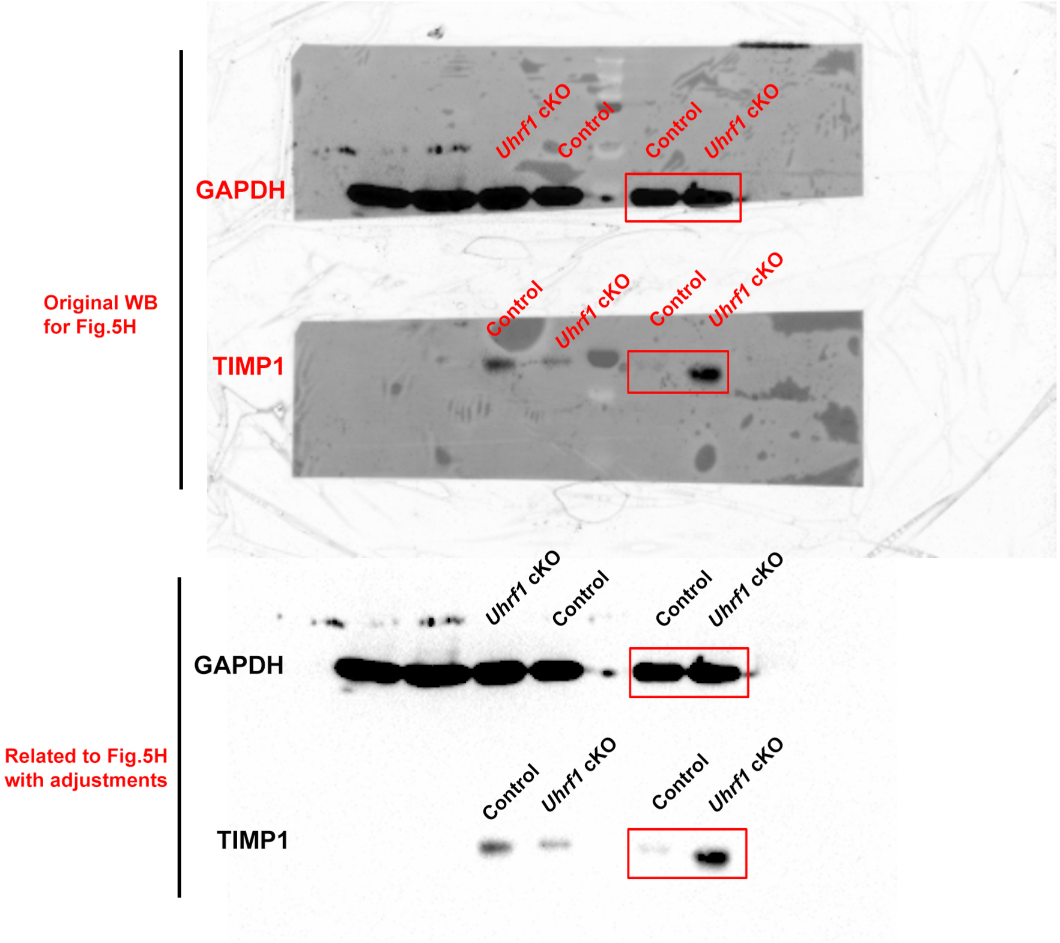

Supplement: Supplementary file 1 — Supplementary Figures [file 41419_2022_4837_MOESM1_ESM.pdf]
